# Diet Overall and Hypocaloric Diets Are Associated With Improvements in Depression but Not Anxiety in People With Metabolic Conditions: A Systematic Review and Meta-Analysis

**DOI:** 10.1016/j.advnut.2024.100169

**Published:** 2024-01-05

**Authors:** Tonya Paris, Robin M Daly, Gavin Abbott, Surbhi Sood, Christine L Freer, Marno C Ryan, Elena S George

**Affiliations:** 1 Institute for Physical Activity and Nutrition (IPAN), School of Exercise and Nutrition Sciences, Deakin University, Geelong, Victoria, Australia; 2 Department of Gastroenterology, St Vincent’s Hospital, Melbourne, Victoria, Australia; 3 Department of Medicine, University of Melbourne Parkville, Melbourne, Victoria, Australia

**Keywords:** anxiety, depression, dietary interventions, metabolic conditions

## Abstract

The risk of depression and anxiety is higher in people with metabolic conditions, but whether dietary approaches, which are central to the management of metabolic conditions, can also improve depression and anxiety is uncertain. The primary aim of this systematic review and meta-analysis was to evaluate the effects of dietary interventions on depression and anxiety in adults with metabolic conditions. The secondary aim was to evaluate the effects of hypocaloric and isocaloric dietary interventions on these outcomes. Four databases (MEDLINE, PsychINFO, EMBASE, and CINAHL) were searched from inception to March 2023. Randomized controlled trials (RCTs) including dietary interventions in adults with metabolic conditions (type 2 diabetes mellitus, hyperlipidemia, hypertension, and/or overweight/obesity) that assessed depression and/or anxiety as outcomes were included. Overall, 13 RCTs were included in the systematic review, ≤13 of which were included in the meta-analysis. Estimates were pooled using random-effect meta-analysis for dietary interventions compared with controls. Improvements in depression scores were found in meta-analytic models including all dietary interventions [pooled estimate for the standardized mean difference (SMD) = −0.20 (95% CI: −0.35, −0.05); *P* = 0.007] and hypocaloric only diets [SMD = −0.27 (95% CI: −0.44, −0.10); *P* = 0.002]. There were no improvements in depression scores with isocaloric dietary interventions only [SMD = −0.14 (95% CI: −0.38, 0.10); *P* = 0.27]. In addition, there were no significant effects of any dietary interventions on anxiety scores. In adults with metabolic conditions, all dietary interventions and hypocaloric diets improved depression, but not anxiety. These findings suggest that dietary interventions including hypocaloric diets can play an important role in the management of depression in people with metabolic conditions.

This systematic review and meta-analysis has been registered with PROSPERO (CRD42021252307).

**Statement of Significance**

Dietary interventions are known to improve metabolic outcomes, which may relate to improvements in depression and anxiety, but whether dietary interventions can improve depression and/or anxiety in people with metabolic conditions is not known. This meta-analysis of 13 RCTs demonstrates, for the first time, that dietary interventions, overall and hypocaloric diets alone, can improve depression but not anxiety in adults with metabolic conditions.

## Introduction

There are an estimated 264 and 322 million people living with anxiety and depression globally, respectively, which have been linked to reduced quality of life [1]. Primary treatment includes psychotherapy and medication, but these treatments do not target other known risk factors such as physical inactivity and poor-quality diet which are common in these individuals [2,3]. Hence, there is a need to include lifestyle strategies to improve the management of depression and anxiety symptoms as well as target-related risk factors such as obesity, which is commonly associated with these mood disorders [[4], [5], [6]].

Metabolic conditions including abdominal or visceral obesity, hypertension, dyslipidemia, and/or glucose dysregulation are estimated to affect 20%–25% of adults globally [[7], [8], [9], [10]] and have been identified as risk factors for both depression and anxiety, with a bidirectional association being implicated [11]. Social factors such as weight stigma, limits to health care access, and low socioeconomic status are also risk factors in the relationship between metabolic conditions and depression and anxiety [12,13]. Those with depression and anxiety have higher rates of metabolic conditions [14,15] and vice versa [[16], [17], [18]]. Multiple interrelated factors have been proposed to underpin the link between these mood disorders and metabolic conditions, including chronic, low-grade systemic inflammation and oxidative stress [19], which are exacerbated by both poor dietary habits and physical inactivity [[20], [21], [22], [23], [24], [25]]. Indeed, there is high-level of evidence that isocaloric and hypocaloric dietary approaches such as low-fat, low-carbohydrate, and the Mediterranean diet can play a role in the management of metabolic conditions [[26], [27], [28], [29]]. Similarly, there is emerging evidence that various dietary patterns (e.g., Mediterranean diet) and some dietary interventions (e.g., low-carbohydrate diet), can help reduce risk and symptoms of depression and anxiety [4,5,[30], [31], [32], [33], [34]]. Whether dietary approaches alone, specifically including hypocaloric or isocaloric dietary interventions, can reduce risk and symptoms of depression and anxiety in those at higher risk with metabolic conditions is unknown.

The primary aim of this systematic review and meta-analysis was to evaluate the effects of dietary interventions on depression and anxiety in adults with metabolic conditions. The secondary aims were to evaluate the effects of isocaloric and hypocaloric dietary interventions separately on depression and anxiety in adults with metabolic conditions.

## Methods

This systematic review was performed based on the PRISMA statement [35] and was registered in the PROSPERO database (CRD42021252307). Several changes were made to the systematic review and meta-analysis after registering with PROSPERO. The aim was expanded from individuals with metabolic syndrome to include those with components of metabolic syndrome given there were insufficient studies with metabolic syndrome only. Additional coauthors (SS, CLF, and GA) were added to assist with specific areas of the review. The inclusion criteria were narrowed to include only randomized controlled trials (RCTs) as there were an extensive number of RCTs identified in the search, and this study design provides a higher level of evidence based on the National Health and Medical Research Council Evidence hierarchy [36]. Regarding “mood,” the outcomes changed to depression and anxiety only. Therefore, the title has been changed along with the primary and secondary aims to specifically reflect that we included adults with metabolic syndrome, or its components, and the focus was specifically on dietary interventions with depression and/or anxiety as outcomes. Additional outcomes extracted include changes in weight to help identify whether improvements were influenced by changes in weight (e.g., weight loss). A meta-analysis was conducted by pooling estimates using random-effect models with subgroup analyses to determine the effects of *1)* hypocaloric and *2)* isocaloric dietary interventions separately on depression and anxiety outcomes.

### Data source

A detailed search was conducted using MEDLINE, PsycINFO, EMBASE, and CINAHL databases from inception to 1 March 2023. English language, humans, and peer-reviewed article filters were applied at the end of each database search. Additional publications were also identified from reference lists of systematic reviews and relevant articles. Details of the search terms used for all the 4 databases can be found in Supplemental Figures 1–4.

### Eligibility criteria

#### Types of studies

Only RCTs were included. Prospective cohort studies, one-arm pilot studies, case–control, cross-sectional, and case-series studies and reviews, letters, editorials, commentaries, animal studies, and duplicate studies were excluded as these study designs do not allow a comparison of the effects of a dietary intervention compared with control.

#### Participants

Studies with participants aged 18 y or older with metabolic conditions, which included metabolic syndrome (defined as 3 of the 5 criteria: obesity, hyperglycemia, dyslipidemia, hypertension) [37], and/or overweight or obesity (BMI ≥ 25 kg/m^2^), and/or prediabetes or type 2 diabetes mellitus (T2DM), and/or hypertension, and/or hyperlipidemia, and/or steatosis, and/or metabolic associated fatty liver disease, and/or nonalcoholic steatohepatitis were included. These metabolic conditions were diagnosed based on the criteria outlined in the respective studies. Participants did not have to be formally diagnosed with clinical depression and/or anxiety to be included. Studies were excluded if they only included healthy weight participants with no metabolic comorbidities, were residents at aged care facilities, inpatients at psychiatric hospitals, or if the study population involved participants with other psychiatric diseases and mental disorders, including schizophrenia, bipolar disorder, post-traumatic stress disorder, eating disorders, attention-deficit/hyperactivity disorder, and obsessive-compulsive disorder.

#### Interventions

Interventions that evaluated the effects of any type of dietary intervention described in sufficient detail were included. Sufficient detail was defined as a “whole of diet approach,” including the main components of the diet such as energy intake and/or food groups and/or macronutrients or interventions with individual or group dietary counseling or nutrition education. All RCTs required a dietary intervention and a control comparison group. Dietary interventions with a combined intervention (e.g., with exercise, stress management, additional dietary supplement(s), cognitive behavioral therapy, pharmaceutical, psychotherapy) were included but only if they contained a diet-only arm and a control group. The control group could include participants following their habitual lifestyle or general information (e.g., a leaflet) about a healthy diet with no prescribed energy recommendations or basic range-of-motion stretches and calisthenic movement.

### Outcome measures

The primary outcome measures were depression and/or anxiety scores, which were derived from validated surveys. Data were extracted if depression and/or anxiety values at baseline and postintervention or mean pre–post change scores or between-group differences in change scores were reported. Authors of relevant studies were contacted (if after reading the full text) baseline and/or postintervention values or mean pre–post change scores were not reported. If these data could not be obtained, the study was excluded.

### Study selection

Publications resulting from the database searches were imported and duplicates were removed using Endnote and Covidence. Screening of title, abstract, and full text were completed using Covidence by 2 independent reviewers (TP and CLF). Studies included after the first screen were read in full, independently by the 2 reviewers (TP and SS) and assessed for eligibility based on the inclusion criteria. Conflicts between reviewers were resolved by a third reviewer (ESG).

### Data extraction and data items

Data extraction for each article in the systematic review was done manually by 1 independent reviewer (TP), which was checked by a second reviewer (SS). Other authors were consulted for clarification on data and outcomes as needed. Data extraction included the following: *1)* participant characteristics (number of participants, sex, study population, age, and the demographic location of the study); *2)* the duration of the intervention and details of the dietary intervention and control group(s); *3)* the method of assessment for depression and/or anxiety scores; *4)* between-group differences in depression and/or anxiety scores and weight with statistical analyses; and *5)* within-group differences in depression and/or anxiety scores and weight with statistical analyses.

### Risk of bias and study methodological quality assessment

The Cochrane Risk of Bias Tool was used to assess risk of bias (high risk, low risk, and unclear) and the studies’ overall quality rating. The Risk of Bias tool version 2.0 [38] assesses bias under the following domains: sequence generation (selection bias), allocation concealment (selection bias), blinding of participants and personnel (performance bias), blinding of outcome assessors (detection bias), incomplete outcome data (attrition bias), selective outcome reporting (reporting bias), and other sources of bias. Risk of bias in all included studies was completed independently by 2 reviewers (TP and SS) for each study. If there were conflicts a third reviewer (ESG) was consulted for an outcome.

### Statistical analysis

For both depression and anxiety, the intervention and control groups were compared on the mean changes from baseline to postintervention for each study. For the primary aim, we included dietary interventions for depression and anxiety separately in adults with metabolic conditions. For the secondary aims, we compared the effect of *1)* only isocaloric dietary interventions on depression and anxiety separately in participants with metabolic conditions, and *2)* only hypocaloric dietary interventions on depression and anxiety in participants with metabolic conditions. Therefore, a total of 6 meta-analyses were conducted. Pooled data were analyzed using a random-effects model in ReviewManager 5. We used random-effects models given that there was no common fixed parameter, studies estimated different parameters, and the interventions were different [39]. Heterogeneity was assessed with the standard Chi-square (significance level: 0.1) and *I*-squared statistics (70%–100% interpreted as considerable heterogeneity, 69%–40% moderate heterogeneity, <40% minor heterogeneity) [40]. Results are presented as standardized mean differences (SMD) with 95% CIs and shown in forest plots. Possible publication bias was examined through visual inspection of funnel plots for all studies in all meta-analyses combined and by technique, the regression-based Egger test for small-study effects, and nonparametric trim-and-fill analysis using Stata/BE 17.0.

## Results

### Study selection

**FIGURE 1:**
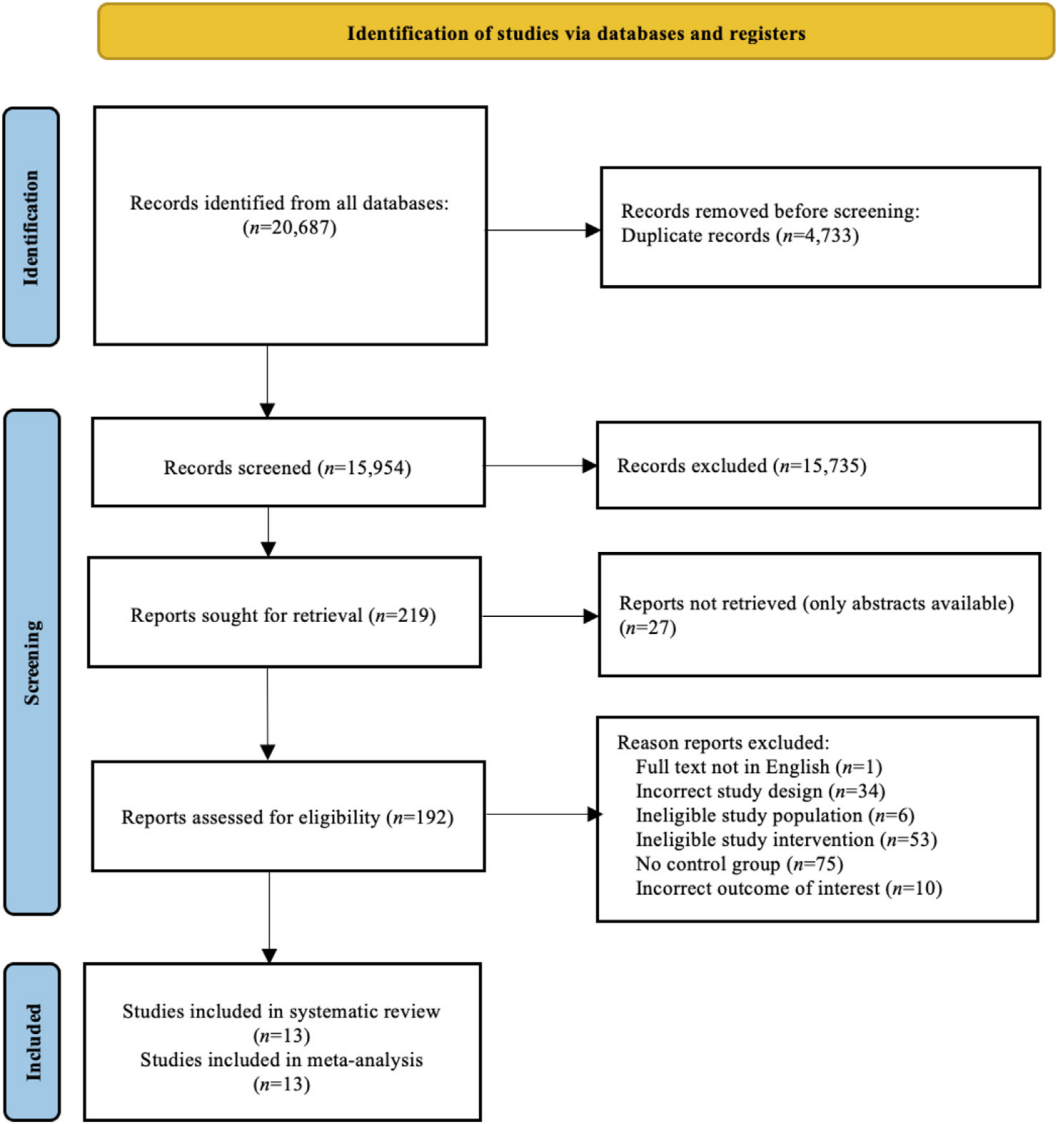
PRISMA flow diagram for the screening, inclusion, and exclusion of studies in this systematic review and meta-analysis.

Four databases (MEDLINE, psychINFO, EMBASE, CINAHL) were searched resulting in a total of 20,687 studies. After removing duplicates 15,954 articles were screened based on title and abstract, from which 219 remained for full-text screening. One study was excluded as the authors only reported baseline depression scores and not postintervention or mean pre–post change score data for depression and did not respond when contacted [41]. Overall, 13 RCTs met the eligibility criteria and were included in the meta-analyses [[42], [43], [44], [45], [46], [47], [48], [49], [50], [51], [52], [53], [54]]. Full PRISMA flow chart of database searches and included studies in the systematic review and meta-analysis are shown in Figure 1.

### Study characteristics

From the 13 included RCTs 2,040 participants were recruited in the studies. Participant numbers for each study ranged from *n* = 44 [53] to *n* = 439 [45]. Over half (*n* = 9, 56%) of the studies included participants with overweight or obesity [[45], [46], [47], [48], [49], [50], [51], [52], [53]] and one study included participants with overweight or obesity and/or previous diagnoses of T2DM [42]. One study included participants with high cholesterol and high blood pressure [43], one study with participants with high blood pressure only [54], and another study with participants with hyperlipidemia [44]*.* Seven studies included a combination of males and females [42,[46], [47], [48],^50^,^51^,^54^], 3 studies included only females [45,49,53], and 3 included only males [43,44,52]. The duration of the interventions ranged from 1 mo [54] to 3 y [43]. Three studies had interventions of 6 mo [44,51,52] and another 3 were of 12-mo duration [45,47,48]. Ten of the 13 studies assessed depression and/or anxiety scores as secondary outcomes [[43], [44], [45],[47], [48], [49], [50], [51], [52],^54^], and 3 studies had depression and/or anxiety as primary outcomes [42,46,53]. Specific details regarding each included study in the systematic review and meta-analysis are described in Table 1 [[42], [43], [44], [45], [46], [47], [48], [49], [50], [51], [52], [53], [54]].

**TABLE 1:** Study characteristics and changes in depression scores, anxiety scores, and weight for the 13 dietary intervention studies included in this review and meta-analysis.

Citation, year, country, duration, RCT design	Number of participants by sex	Population group and age (y)	Diet and control groups	Depression and/or anxiety assessment	Between-group differences	Within-group differences
Agarwal et al., 2015 [42], United States, 4.5 mo, parallel design	233 F 49 M	Participants with overweight or obesity (BMI ≥25 kg/m^2^) and/or previous diagnoses of T2DM Age (mean and SD): Diet group: 43.8 ± 10.6 Control group: 45.4 ± 11.3	Diet group: *n* = 136 Low-fat vegan diet • Fruits, vegetables, whole grains, and legumes • Avoid animal products • Minimal added oils, < 3 g of fat a serve • Consume low GI foods • B12 supplement • Intervention sites with cafes provide low-fat vegan meals and snacks • No restrictions on portion sizes, energy, or CHO intake • 1-h education on GI index, foods to favor and avoid • Weekly hour nutrition group sessions on weight loss and chronic diseases, cooking demonstrations, and discussion Control group: *n* = 145 • Habitual lifestyle • Same contact time as the diet group • Participants were compensated with a $50 gift voucher	SF-36 Depression and anxiety subscales	ITT analysis + Depression (unadjusted) ↔ Depression (adjusted^1^) + Anxiety (unadjusted) ↔ Anxiety (adjusted^1^) Weight not reported	ITT analysis Diet group + Depression + Anxiety Control group ↔ Depression ↔ Anxiety Weight not reported
Einvik et al., 2010^2^ [43], Norway, 3 y, factorial design	505 M	Participants with high cholesterol (>6.45 mmol/L) and systolic blood pressure <150 mmHg Age (range): No diet group: 70 mean (64–75) Diet group: 70 (65–75)	2 × 2 Factorial placebo-controlled study of n-3 PUFA and/or dietary counseling Diet group: *n* = 139 Diet counseling and placebo • Increase vegetables, fruit, and fish • Decrease meat and fat from animals • Increase vegetable oils and margarines (rapeseed oil, olive oil, and sunflower oil) • Special oil and margarine were supplied at all visits • Adopt a calorie-restricted diet (if overweight or obese) • Dietary counseling by a nutritionist based on FFQ for 30–45 min at baseline and after 6 mo • Visits with nutritionist every 6 mo for the remaining study Control group and placebo: *n* = 142 • Habitual lifestyle and placebo (corn oil) • Same contact time as the diet group	HADS	ITT analysis ↔ Depression ↔ Anxiety ↔ Weight loss^3^	ITT analysis Diet group *n* = 253 − Depression − Anxiety No diet group *n* = 252 − Depression − Anxiety Diet group and placebo ↔ BMI Control group ↔ BMI
Hyyppä et al., 2003 [44], Finland, 6 mo, crossover design	120 M	Participants with untreated hypercholesterolemia aged between 35 and 64 y and a fasting serum cholesterol level between 6.0 and 8.0 mmol/L Age (mean and SD): control group + placebo/simvastatin 48.4 ± 6.2 Diet group+placebo/simvastatin 48.0 ± 6.2	A randomized double-blind placebo-controlled crossover trial with separate and combined effects of a Mediterranean diet and treatment with simvastatin 20 mg/d PO Diet group: *n* = 60 Mediterranean diet and placebo • ≤10% E intake from SFA and trans-unsaturated fatty acid • Cholesterol intake ≤ 250 mg/d • Omega-3 fatty acid intake of plant and marine origin of ≥4 g/d and the ratio of omega-6/omega-3 PUFA < 4 • Increase fruits, vegetables, and soluble fiber • Lean meats and low-fat dairy • Fish 1–2/wk • Consume rapeseed over butter • Rapeseed margarine and oil, oat bran, and frozen berries supplied • One individual and 2 group counseling sessions at baseline and in 5 subsequent monthly group sessions with nutritionist Control group and placebo: *n* = 60 • Habitual lifestyle and placebo (wheat starch and additives)	BSI-18 depression and anxiety subscales	Analysis not reported Depression, anxiety, weight not reported	Analysis not reported Diet group ↔ Depression ↔ Anxiety ↔ Weight Control group Depression and anxiety not reported ↔ Weight
Imayama et al., 2011 [45], United States, 1 y, parallel design	439 F	Participants with overweight or obesity (BMI ≥ 25.0 kg/m^2^ (if Asian-American ≥ 23.0 kg/m^2^)) females that are postmenopausal aged 50–75 y old Age (mean and SD): Diet group: 58.1 ± 5.9 Control group: 57.4 ± 4.4	Diet group: *n* = 118 Hypocaloric diet • 1200–2000 kcal/d based on baseline weight • ≤30% E intake from fat • 10% weight loss within first 24 wk with maintenance for rest of the trial period • Individual visits with dietitian for personalized goal setting on 2 occasions, then weekly meetings through the first 6 mo. After 6 mo, dietitians made contact twice a month (1 face-to-face contact and 1 additional contact via phone or email) • Sessions develop strategies and skills, calorie and weight loss goals by self-monitoring, goal setting, coping strategies, and problem solving Control group: *n* = 87 • Habitual lifestyle	BSI-18 depression and anxiety subscales	ITT analysis ↔ Depression^4^ ↔ Anxiety^4^ + Weight loss	ITT analysis Diet group ↔ Depression^5^ ↔ Anxiety^5^ + Weight loss Control group ↔ Depression^5^ ↔ Anxiety^5^ ↔ Weight loss
Jenkinson et al., 2009 [46], United Kingdom, 2 y, factorial design	257 F 132 M	Participants with overweight and obesity (BMI ≥ 28.0 kg/m^2^) with knee pain, > 45 y old Age (mean and SD): Diet group: 61.7 ± 9.2 Control group: 61.5 ± 9.2	Factorial study with 4 groups; diet, diet + exercise, exercise, and a control group Diet group: *n* = 122 Hypocaloric diet • Individual diet advice with a calorie deficit of 2.5 MJ/d • Reduce fat, sugar, and portions • More fruit and vegetables • Weight loss of 0.5–1.0 kg a week • Monthly home visits for the first 6 mo and then every other month for the duration of the 2-y follow-up • Newsletters, recipes, and healthy eating advice were sent every few months by a dietitian Control group: *n* = 76 • Advice leaflet given on the Arthritis Research Campaign leaflet for knee osteoarthritis, but intervention information was removed • Home visits conducted every 4 mo and support telephone calls in between their visits • Participants were asked about their knee pain, general health, medications, and physical activity	HADS	ITT analysis Not reported in depression and anxiety + Weight loss	ITT analysis Diet group *n* = 231 + Depression ↔ Anxiety Exercise group *n* = 191 ↔ Depression ↔ Anxiety Weight not reported
Kiernan et al., 2001 [47], United States, 1 y, parallel design	112 F 119 M	Males with overweight (BMI of 28–34 kg/m^2^) and females with overweight and premenopausal (BMI of 24–30 kg/m^2^) Age (mean and SD): 38.5 ± 6.4	Diet group: *n* = 71 Low-fat, SFA, and cholesterol diet • <30% E intake from fat • <10% E intake from SFA • <300 mg/d cholesterol • Maintenance calories • ≤10% E intake from PUFA • 10%–15% E intake from MUFA • 50%–60% E intake from CHO • Weekly classes by dietitians for the first 3 mo, every other week for the next 3 mo, and monthly for the remaining 6 mo Control group: *n* = 79 • Habitual lifestyle	BDI Taylor Manifest Anxiety Scale	Analysis not reported Not reported in depression and anxiety + Weight loss	Analysis not reported Diet group ↔ Depression ↔ Anxiety + Weight loss Control group ↔ Depression ↔ Anxiety ↔ Weight loss
Napoli et al., 2014 [48], United States, 1 y, parallel design.	67 F 40 M	≥65 y old participants with obesity (BMI ≥ 30 kg/m^2^) Age (mean and SD): Diet group: 70 ± 4 Control group: 69 ± 4	Diet group: *n*=26 Hypocaloric diet • Energy deficit of 500–700 kcal/d from requirements • 1 g Protein per kg/d • Weekly group sessions with dietitian to adjust caloric intake and behavioral therapy, goals, and weigh-ins • 10% loss of body weight at 6 mo and maintain it Control group: *n* = 27 • General information only on healthy diet at monthly visits • Did not participate in the diet or exercise regimes	GDS	ITT analysis ↔ Depression + Weight loss	ITT analysis Diet group ↔ Depression + Weight loss Control group ↔ Depression ↔ Weight loss
Nieman et al., 2000 [49], United States, 3 mo, parallel design	91 F	Participants with obesity (BMI of 25–50 kg/m^2^) Age (mean and SE): 45.6 ± 1.1	Diet group: *n*=26 Hypocaloric diet • 1200–1300 kcal/d • Meal plan based on diet exchanges: 2 fruit, 3 vegetables, 2 milk, 6 bread, 2 fat, 5 lean protein, and 100 kcal optional foods • Dietitian education on portion sizes, food exchanges and recording intake • Weekly instructions on weight loss principles and nutrition guidelines • 4 days/week 45-min stretching and mild range-of-motion calisthenic exercises, heart rate below 100 beats/minute Control group: *n* = 22 • Stretching and calisthenic exercises as per the diet group • Same contact time as diet group	POMS (global score)	Analysis not reported ↔ Depression + Weight loss	Analysis not reported Diet group ↔ Depression + Weight loss Control group ↔ Depression ↔ Weight loss
Özbey-Yücel et al., 2023 [50], Turkey, 3 mo, parallel design	43 F 20 M	Participants with obesity and tinnitus (BMI > 30 kg/m^2^) Age (mean and SD): Diet group: 46 ± 11.3 Control group: 43.1 ± 8.1	Diet group: *n* = 16 Hypocaloric diet • Monitoring by phone every 2 wk to correct and maintain diet • Energy requirements calculated by the Schofield equation using age and sex • 10%–20% E intake from protein • 45%–60% E intake from CHO • 20%–35% E from fat • Track daily steps Control group: *n* = 17 • Track daily steps • Same contact time as the diet group	BDI	Analysis not reported + Depression + Weight loss	Analysis not reported Diet group + Depression + Weight loss Control group + Depression ↔ Weight loss
Senna et al., 2012 [51], Egypt, 6 mo, parallel design	75 F 8 M	Participants with obesity (BMI ≥ 30 kg/m^2^) and fibromyalgia Age (mean and SD): Diet group: 44.8 ± 13.6 Control group: 46.3 ± 14.4	Diet group: *n* = 41 Hypocaloric diet • 1200 kcal/d • 15%–20% E intake from protein • 50%–55% E intake from CHO • 30% E intake from fat • Food options were vegetables, fruits, wholegrain, and low-fat dairy • Instructed on accurate recording of dietary intakes and reviewed during monthly visits • Sample meal plan and recipes • Follow medical treatment advised by physician Control group: *n* = 42 • Follow medical treatment advised by physician • Followed the same healthy food and ratio of each component as per the diet group without restriction in calories	BDI-II	Analysis not reported + Depression + BMI	Analysis not reported Diet group + Depression + BMI Control group ↔ Depression ↔ BMI
Tan et al., 2016 [52], Finland, 6 mo, parallel design	49 M	Participants with overweight or obesity (BMI ≥ 25 kg/m^2^) and chronic sleep insomnia symptoms Age (mean and 95% CI): Diet group: 51.0 (47.3–54.8) Control group: 52.6 (48.0–57.2)	Diet group: *n* = 28 Hypocaloric diet • Reduce and maintain calories by 300–500 kcal/d first 3 mo • 40%–45% E intake from CHO • 35%–40% E intake from fat with ≤10% E intake from SFA • 15%–20% E intake from MUFA, 5%–10% intake E from PUFA • 20% E intake from protein. • Increase fiber, vitamin A, D, E, B, C, Mg, and K • Reduce weight by 3 kg • Two intermediate face-to-face counseling sessions held in the first and fourth month • Each intermediate session had individualized diet counseling with a nutritionist and a cooking course • Online counseling tracker to assess and provide feedback on dietary intake Control group: *n* = 21 • Habitual lifestyle	Rimon’s depression scale	ITT analysis ↔ Depression^6^ + Weight loss	ITT analysis Diet group + Depression + Weight loss Control group ↔ Depression − Weight loss
Uemura et al., 2019 [53], Japan, 2 mo, parallel design	44 F	Participants with obesity (BMI ≥ 25 kg/m^2^ or waist circumference ≥ 90 cm) Age (mean and SD): Diet group: 62.0 ± 8.7 Control Group: 63.3 ± 9.1	Diet group: *n* = 22 Food and nutrients for the microbiome • Association between the gut microbiome and obesity/depression • Relationship between the gut microbiome and dietary habits • Optimal dietary intake referring to the Japanese food guide Spinning Top and recipes • Education on the gut microbiome and fermented foods, fiber foods, fiber goals, fermented food goals, and fiber content in food. • 20-min education lectures and 10-min individual counseling every 2 wk • Self-management of diet habits, ≤3% weight reductions, <3 cm for waist circumference • Set program goals, and achievement level was monitored during each visit • Program goals based on food and nutrient intake, decreases in weight and waist circumference postintervention Control group: *n* = 22 • Habitual lifestyle	CES-D	Analysis not reported + Depression + Weight loss^7^	Analysis not reported Diet group + Depression + Weight loss^7^ Control group ↔ Depression ↔ Weight loss^7^
Yau et al., 2022 [54], China, 1 mo, parallel design	55 F 17 M	Participants with stage 1 (systolic blood pressure 130–139 mmHg or diastolic blood pressure 80–89 mmHg) or stage 2 HTN (systolic blood pressure ≥140 mmHg or diastolic blood pressure ≥90 mmHg) Age: 66.9 ± 9.7 y	Diet group: *n* = 23 Mediterranean DASH diet • One session with a nutritionist, 1 h face-to-face or online mode to modify their diet • Sodium restriction (<1500 mg/d) • Potassium intake (3500–5000 mg/d). • Increased olive oil, fish and poultry, nuts, and berries, fruits, low-fat dairy, wholegrains, legumes/pulses, ≥ 3 servings of vegetables and green vegetables • Low intake of fat and SFA, red meats, processed wholegrains, eggs, and added sugars • Nutritionists guiding how often the recommended foods should be consumed • Discussion for establishing rapport with participants • Blood pressure measurement • Dietary adoption and adherence to nutritional recommendation Control group: *n* = 24 • Habitual lifestyle	POMS- depression-dejection STAI-T	ITT analysis ↔ Depression ↔ Anxiety ↔ Weight loss	ITT analysis Diet group ↔ Depression ↔ Anxiety ↔ BMI Control group ↔ Depression ↔ Anxiety ↔ BMI

Abbreviations: BDI, Beck’s Depression Inventory; BSI-18, Brief Symptom Inventory-18; CHO, carbohydrates; CES-D, Centre for Epidemiologic Studies Depression; DASH, dietary approaches to stop hypertension; E, energy; FA, fatty acids; FFQ, food frequency questionnaire; GDS, geriatric depression scale; GI, glycemic index; HADS, hospital anxiety and depression rating scale; HTN, hypertension; ITT, intention-to-treat; PO, orally by mouth; POMS, profile of mood states; RCT, randomized controlled trials; SF-36, Short Form-36; STAI, State-Trait Anxiety Inventory; T2DM, type 2 diabetes mellitus.

Results represented by + = significantly decreased (improvement) in the intervention relative to controls, - = significantly increased (worsened) in the intervention relative to controls, ↔ = no significant difference in the intervention relative to controls.

1 Adjusted for sex, cluster, medications, and baseline values in univariate analysis.

2 In the study by Einvik et al., 2010 [43], the Oslo diet and Antismoking Study from 1972 to 1977 where all participants that received traditional lifestyle advice, including advice on cessation of smoking, and half the participants were randomly assigned to dietary counseling (dietary counseling *n* = 604 compared with no diet counseling *n* = 628). The study relevant to this review is the 25-y follow-up (DOIT) study with *n* = 505 male participants from 1997 to 2003.

3 Analysis of covariance with adjustment for baseline values examining independent effects of diet.

4 Adjusting for the baseline scores and covariates (depression: medication use, anxiety: medication use).

5 Adjusted means are changes in psychological factors adjusted for baseline scores and covariates (e.g., age, baseline BMI, marital status, anxiolytics, and antidepressant use).

6 Controlling for baseline values.

7 Adjusted for energy intake as a covariate.

### Characteristics of the dietary interventions and control group

#### Dietary intervention group characteristics

Dietary interventions differed across all the included studies. Four studies provided nutrition education and counseling [42,43,53,54], with topics including following a low-fat vegan diet [42], a plant-based diet [43], high-fiber foods and nutrients for the microbiome [53], or a Mediterranean Dietary Approaches to Stop Hypertension (DASH) for neurodegenerative delay [54]. The DASH diet promotes sodium restriction and an increase in potassium intake, increasing fruits, vegetables, whole grains, low-fat dairy, lower saturated and total fat with a Mediterranean diet high in olive oil, green vegetables, fruits, moderate fish and other meats, cereals, nuts, legumes, dairy products, low intake of eggs, red wine, and sweets [54].

Two studies focused on specific macronutrient targets and food groups without a set calorie goal [44,47]. One study prescribed a Mediterranean diet [44], and another maintenance of calories and goals for fat, cholesterol, and carbohydrates (CHO) [47]. Seven studies encouraged a specific calorie deficit only or caloric deficit and macronutrient goals [45,46,[48], [49], [50], [51], [52]]. Two studies had set calorie goals for all participants (1200–1300 kcal/d) [49,51] and 5 studies calculated each participant’s baseline energy/calorie requirements and provided an energy/calorie deficit [45,46,48,50,52].

#### Control group characteristics

Five of the 13 control groups had participants who continued their habitual lifestyle [42,45,47,[52], [53], [54]]. Two studies had control participants continuing their habitual lifestyle with a placebo, one with wheat starch and additives [44] and the other with corn oil [43]. One provided an advice leaflet based on the Arthritis Research Campaign (United Kingdom) leaflet for osteoarthritis of the knee, (information related to the intervention removed) and home visits conducted every 4 mo where participants were asked about their general wellbeing and lifestyle, with additional support via phone calls between home visits [46]. Another did not receive lifestyle advice or participate in the diet and exercise intervention group regimes and were given general information about a healthy diet during monthly visits with researchers [48]. One prescribed 45-min sessions of stretching 4 d/wk and range-of-motion calisthenic exercises to keep heart rate below 100 beats/min [49], whereas another had participants track their daily steps [50], and one study followed the same healthy food and ratio of each component as per the dietary group without calorie restriction [51].

Four of 13 studies had matched the dietary intervention and control groups for researcher contact time [42,43,49,50]. Nine of 13 studies did not match the dietary intervention and control groups for researcher contact time [[44], [45], [46], [47], [48],[51], [52], [53], [54]].

Specific details regarding dietary intervention and control group and matching groups in each included study in the systematic review and meta-analysis can be found in Table 1 [[42], [43], [44], [45], [46], [47], [48], [49], [50], [51], [52], [53], [54]].

#### Dietary adherence and/or dietary intake

Dietary adherence and/or dietary intake methods and results varied considerably across all studies. Three of the 13 studies did not report dietary adherence and/or intake [46,48,51]. In 8 of the 13 studies that reported dietary intake, the dietary intervention group overall showed reductions in energy [45,47,49,50,52], fat, SFA, and cholesterol [[43], [44], [45],^47^,^50^], CHO [50], PUFA [50], MUFA [43], protein [50], and increases in fiber [43,44,53], CHO [43], dairy [53], vegetables [53], vitamins [44,52], PUFA [43,44], and MUFA [44] intake. One of 10 studies defined adherence to the vegan low-fat diet as ≤35% of the total energy intake from fat and cholesterol intake of ≤75 mg/d, with 85% of the dietary group and 21% of the control group as adherent to the vegan component (*P* < 0.001) and 86% of the dietary group compared with 40% of the control group as adherent to the low-fat component of the diet (*P* < 0.001) [42]. One of the 10 studies used the Mediterranean DASH intervention for neurodegenerative delay score at baseline and after the intervention, with a maximum score of 15 indicating the highest level of adherence [54]. The mean score significantly increased by 3.8 in the dietary group, with baseline and postintervention scores not reported [54].

Specific details regarding dietary adherence and/or dietary intake in each included study in the systematic review and meta-analysis can be found in Supplemental Table 1.

### Overall study findings for depression and anxiety

#### Depression

All 13 studies included depression as an outcome [[42], [43], [44], [45], [46], [47], [48], [49], [50], [51], [52], [53], [54]], which was assessed using different instruments: Beck Depression Inventory (BDI) (*n* = 3) [47,50,51], Hospital Anxiety and Depression Rating Scale (HADS) (*n* = 2) [43,46], Brief Symptom Inventory-18 (BSI-18) (*n* = 2) [44,45], Profile of Mood States (POMS) (*n* = 2) [49,54], Geriatric Depression Scale (GDS) (*n* = 1) [48], Short Form-36 (SF-36) (*n* = 1) [42], Rimon’s Depression Scale (*n* = 1) [52], and Centre for Epidemiologic Studies Depression (CES-D) (*n* = 1) [53]. Two of the 13 studies had combined groups into “diet” and “no diet” rather than having results presented as “diet groups” and “control groups” as they were factorial studies [43,46].

Of the 7 studies with hypocaloric dietary interventions [45,46,[48], [49], [50], [51], [52]], all reported significant between-group differences in weight loss in favor of the dietary intervention [45,46,[48], [49], [50], [51], [52]]. Three of the 7 studies reported no significant between-group difference in depression change scores [45,48,49]. The length of the intervention ranged between 3 mo and a set hypocaloric dietary intervention of 1200–1330 kcal/d [49] to 1 y [45,48] with a total daily energy intake of 1200–2000 kcal/d based on baseline weight [45] and a 500–700 kcal/d calorie deficit based on calculated calorie requirements [48]. Two of the 7 studies reported significant differences between groups in favor of the dietary group for depression change scores [50,51], with the length of the interventions being a 3-mo hypocaloric dietary intervention calculated for each participant using the Scholfield equation [50] and a 6-mo 1200 kcal/d hypocaloric dietary intervention [51]. One of 7 studies did not report data on differences between groups in depression change score, yet reported a significant within-group decrease in depression change score in the dietary group after a 2-y hypocaloric dietary intervention deficit of 2.5 MJ/d based on calculated requirements [46]. One of 7 studies reported no significant between-group differences in depression change score, yet there was a significant within-group difference in depression change score in the dietary group after a 6-mo hypocaloric dietary intervention with a deficit of 300–500 kcal/d based on energy requirements [52].

Of the 6 studies with isocaloric dietary interventions [[42], [43], [44],^47^,^53^,^54^], 2 reported a significant difference in weight loss between the groups in favor of the dietary group [47,53], with only 1 study showing significant group differences for depression change scores in favor of the dietary group (a 2-mo education on food and nutrients for the microbiome intervention) [53]; the other study did not report group differences in depression change score, however, there was no significant within-group improvement in depression change score in the dietary group after a 1-y low-fat, SFA, and cholesterol dietary intervention [47]. One study did not report data on weight but reported a significant group difference in depression change scores in favor of the dietary group after 4 and a half months of a low-fat vegan dietary intervention [42]. One study reported no significant difference between groups in depression change score and weight loss, however, there was a significant within-group increase (worsening) in depression change score after a 3-y plant-based counseling intervention [43]. One study reported no significant within or between-group differences for changes in weight or depression scores after a 1 mo Mediterranean DASH dietary intervention [54], and 1 study did not report data for the between-group differences for changes in weight or depression score, however, there was no significant within-group differences for changes in weight or depression scores after the 6-mo Mediterranean dietary intervention [44].

#### Anxiety

Seven of the 13 studies included anxiety as an outcome [[42], [43], [44], [45], [46], [47],^54^], which was assessed using several different instruments: HADS (*n* = 2) [43,46], BSI-18 (*n* = 2) [44,45], State-Trait Anxiety Inventory (STAI) (*n* = 1) [54], SF-36 (*n* = 1) [42], and Taylor Manifest Anxiety Scale (*n* = 1) [47]. Two of the 7 had combined group into “diet” and “no diet” rather than having results presented as “diet groups” and “control groups” as they were factorial studies [43,46].

There were 2 studies with hypocaloric dietary interventions [45,46]. Both studies reported significant between-group differences in weight loss favoring the dietary intervention [45,46], however, one study reported no significant between-group difference for anxiety change score after a 1-y hypocaloric dietary intervention with a total daily energy intake of 1200–2000 kcal/d based on baseline weight [45], and the other study reported no significant within-group difference in anxiety change score as data for between-group differences was not reported after a 2-y hypocaloric dietary intervention deficit of 2.5 MJ/d based on calculated requirements [46].

Of the 5 isocaloric dietary interventions, 1 study reported (after a 1-y low-fat, SFA, and cholesterol dietary intervention) that there was a significant between-group difference for weight loss favoring the dietary group, but no significant within-group difference for anxiety change score, as between-group data were not reported [47]. One of 5 studies did not report data on weight after a 4-and-a-half month low-fat vegan dietary intervention, however, there was a significant between-group difference in anxiety change score favoring the dietary intervention [42]. One of 5 studies reported no significant between- and within-group differences in weight loss and anxiety change scores after a 1-mo Mediterranean DASH dietary intervention [54]. One of 5 studies reported no significant between-group difference weight loss and anxiety change scores, however, there was a significant within-group increase (worsening) in anxiety change scores and no change in weight after a 3-y plant-based dietary counseling intervention [43]. One study reported no significant within-group differences in weight loss and anxiety change scores in a 6-mo Mediterranean dietary intervention, as data for between-group differences were not reported [44].

Specific details regarding changes in depression scores, anxiety scores, and weight from each included study in the systematic review and meta-analysis can be found in Table 1 [[42], [43], [44], [45], [46], [47], [48], [49], [50], [51], [52], [53], [54]] and Supplemental Table 1.

### Meta-analyses for depression and anxiety

#### Effects of dietary interventions on depression scores

**FIGURE 2:**
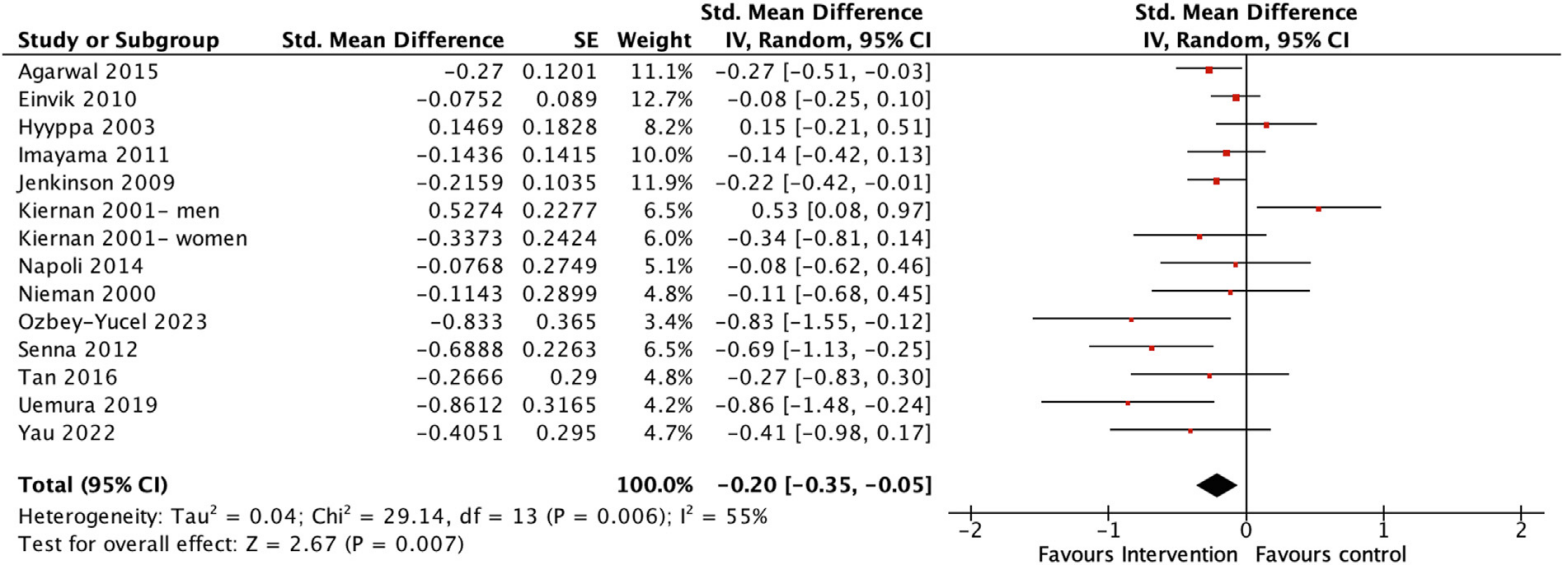
Forest plot assessing differences in change in depression score between baseline and postintervention between the dietary intervention and control group. CI, confidence interval; df, degrees of freedom; Std, standardized.

All 13 studies including 2040 participants investigated the effects of dietary interventions on depression scores and were included in the meta-analysis [[42], [43], [44], [45], [46], [47], [48], [49], [50], [51], [52], [53], [54]]. Overall, the pooled estimate indicated a significant beneficial effect of the dietary interventions on depression scores (SMD = −0.20; 95% CI: −0.35, −0.05, *P* = 0.007) (Figure 2), with moderate heterogeneity among the 13 studies (*χ*^2^ = 29.14, *I*^2^ = 55%, *P* = 0.006).

#### Effects of dietary interventions on anxiety scores

**FIGURE 3:**
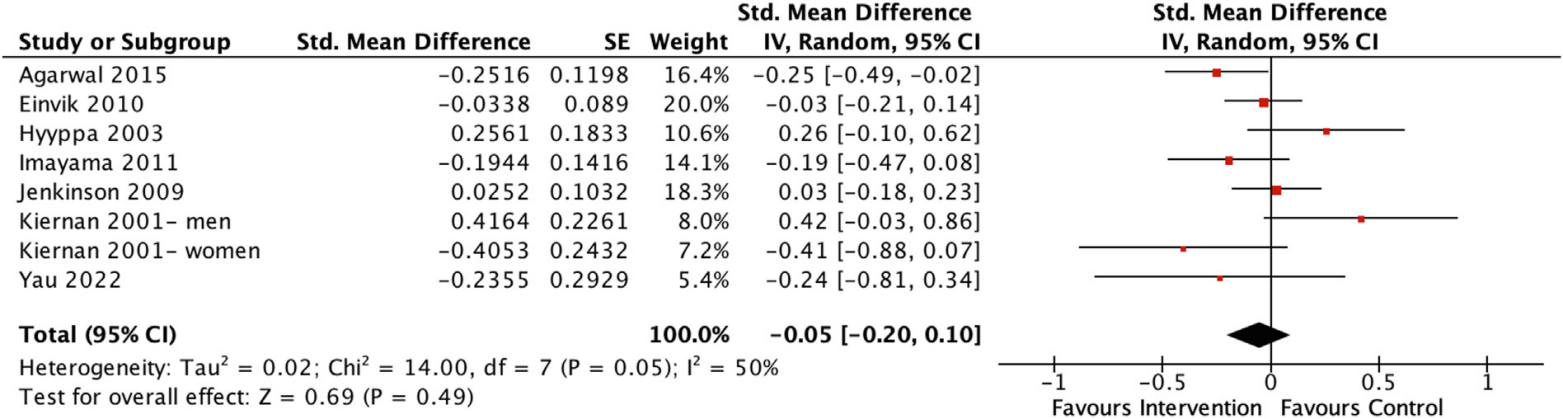
Forest plot assessing differences in change in anxiety score between baseline and postintervention between the dietary intervention and control group. CI, confidence interval; df, degrees of freedom; Std, standardized.

Seven studies including 1730 participants investigated the effects of dietary interventions on anxiety scores [[42], [43], [44], [45], [46], [47],^54^]. Overall, the pooled estimate indicated no significant effect of the dietary interventions on anxiety scores (SMD = −0.05; 95% CI: −0.20, 0.10), *P* = 0.49) (Figure 3), with moderate heterogeneity among the 7 studies (*χ*^2^ = 14.00, *I*^2^ = 50%, *P* = 0.05).

#### Effects of hypocaloric dietary interventions on depression scores

**FIGURE 4:**
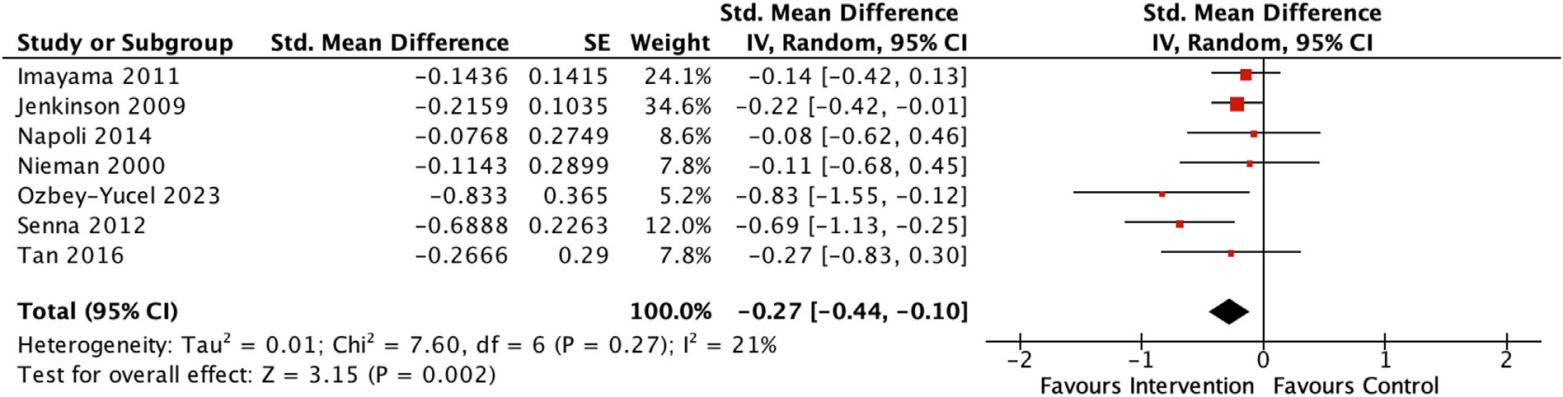
Forest plot assessing differences in change in depression score between baseline and postintervention between the hypocaloric diet intervention and control group. CI, confidence interval; df, degrees of freedom; Std, standardized.

Seven studies including 893 participants were included in the meta-analysis that investigated only hypocaloric dietary interventions on depression scores [45,46,[48], [49], [50], [51], [52]]. Overall, the pooled estimate indicated a statistically significant beneficial effect of hypocaloric dietary interventions on depression scores (SMD = −0.27; 95% CI: −0.44, −0.10, *P* = 0.002) (Figure 4), with minor heterogeneity among the 7 studies (*χ*^2^ = 7.60, *I*^2^ = 21%, *P* = 0.27).

#### Effects of hypocaloric dietary interventions on anxiety scores

**FIGURE 5:**
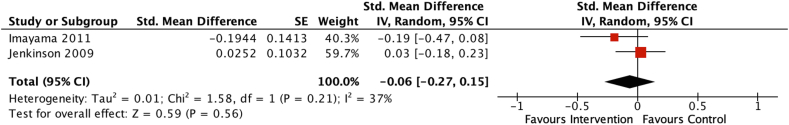
Forest plot assessing differences in change in anxiety score between baseline and postintervention between the hypocaloric diet intervention and control group. CI, confidence interval; df, degrees of freedom, Std; standardized.

Two studies with 627 participants were included in the meta-analysis investigating the effects of hypocaloric dietary interventions on anxiety scores [45,46]. Overall, the pooled estimate indicated no significant benefit of hypocaloric dietary interventions on anxiety scores (SMD = −0.06; 95% CI:−0.27, 0.15), *P* = 0.56) (Figure 5), with minor heterogeneity among the 2 studies (*χ*^2^ = 1.58, *I*^2^ = 37%, *P* = 0.21).

#### Effects of isocaloric dietary interventions on depression scores

**FIGURE 6:**
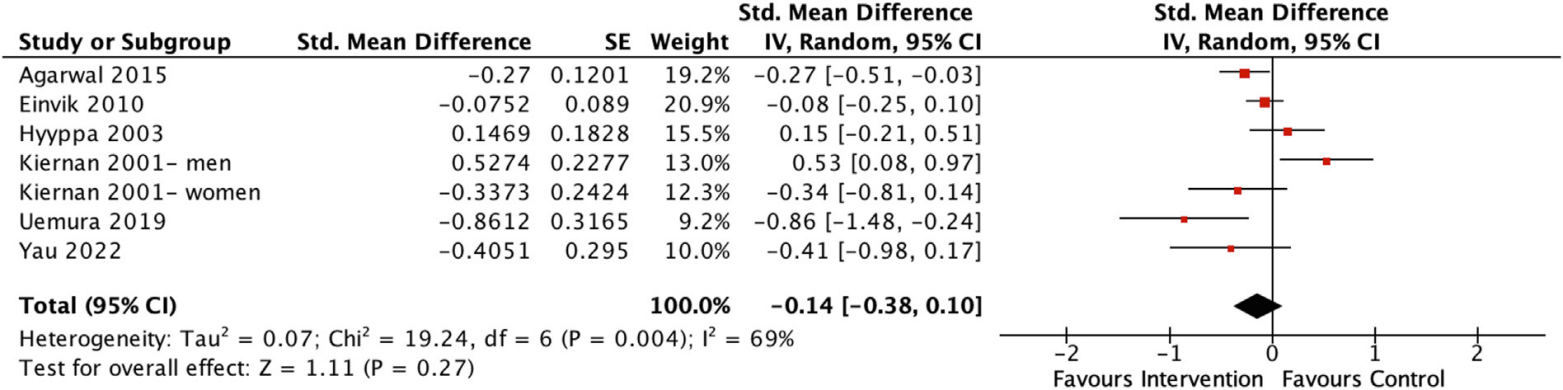
Forest plot assessing differences in change in depression score between baseline and postintervention between the isocaloric diet intervention and control group. CI, confidence interval; df, degrees of freedom; Std, standardized.

Six studies with 1147 participants were included in the meta-analysis investigating the effects of isocaloric dietary interventions on depression scores [[42], [43], [44],^47^,^53^,^54^]. Overall, the pooled estimate indicated no significant benefit of isocaloric dietary interventions on depression scores (SMD = −0.14; 95% CI: −0.38, 0.10, *P* = 0.27) (Figure 6), with moderate heterogeneity among the 6 studies (*χ*^2^ = 19.24, *I*^2^ = 69%, *P* = 0.004).

#### Effects of isocaloric dietary interventions on anxiety scores

**FIGURE 7:**
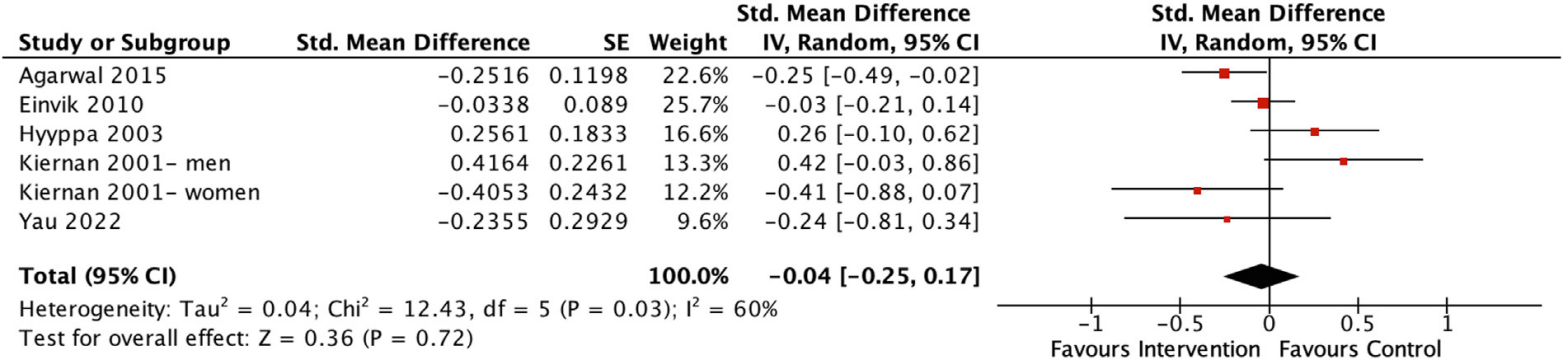
Forest plot assessing differences in change in anxiety score between baseline and postintervention between the isocaloric diet intervention and control group. CI, confidence interval; df, degrees of freedom; Std, standardized.

Five studies with 1103 participants were included in the meta-analysis investigating the effects of isocaloric dietary interventions on anxiety scores [[42], [43], [44],^47^,^54^]. Overall, the pooled estimate indicated no significant benefit of isocaloric dietary interventions on anxiety scores (SMD = −0.04; 95% CI: −0.25, 0.17, *P* = 0.72) (Figure 7), with moderate heterogeneity among the 5 studies (*χ*^2^ = 12.43, *I*^2^ = 60%, *P* = 0.03).

### Risk of bias

**FIGURE 8:**
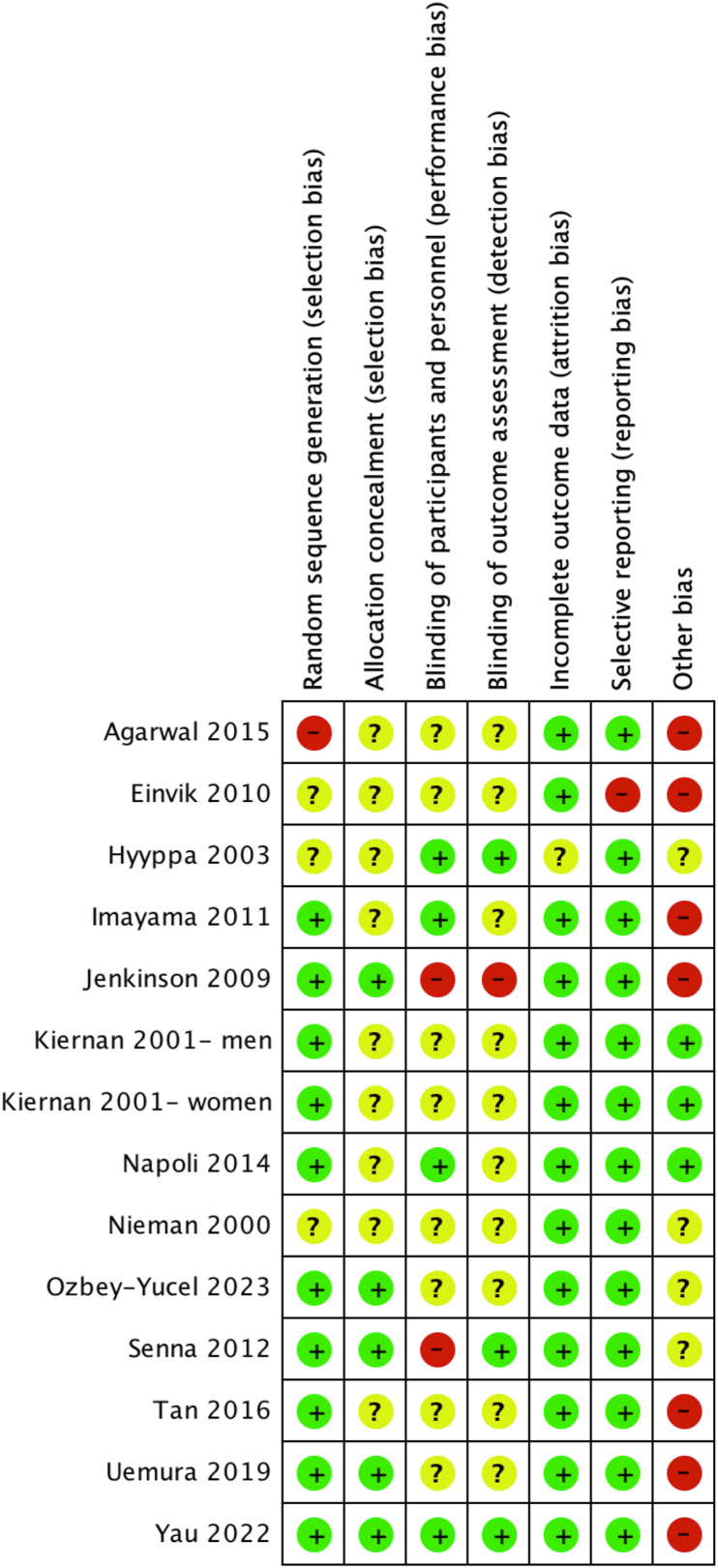
Risk of bias assessment using the Cochrane Risk of Bias Tool Version 2 for all included studies in this systematic review. Green, low risk; yellow, unclear risk; red, high risk.

Figure 8 shows risk of bias assessment for all 13 studies with risk of bias graph included in Supplemental Figure 5. Using the Cochrane Risk of Bias Tool Version 2 [38], 10 studies obtained an overall result of “poor quality,” which was due to >2 criteria scoring “high risk” or “unclear” [[42], [43], [44], [45], [46], [47],^49^,^50^,^52^,^53^]. Three studies obtained an overall result of “fair quality” because they scored “unclear”/“high risk” on 2 criteria [48,51,54]. Potential publication bias was first checked via examination of funnel plots. Funnel plot asymmetry was not evident.

The regression-based Eggers test produced nonsignificant results for all meta-analyses, indicating little evidence of small-study effects. However, the trim-and-fill analyses indicated potential publication bias in 3 of the meta-analyses examining: *1)* dietary interventions on depression scores, *2)* hypocaloric dietary interventions on anxiety scores, and *3)* isocaloric dietary interventions on depression scores, with pooled estimates including imputed studies showing a statistically significant difference in the original pooled estimate compared with the trim-and-fill analysis. Full analysis of all funnel plots and trim-and-fill analyses for each meta-analysis is reported in Supplemental Figures 6–11.

## Discussion

This systematic review and meta-analysis is the first to our knowledge, to comprehensively evaluate the effects of dietary interventions on depression and anxiety in adults with common metabolic conditions. Our findings indicate that there was a beneficial effect of all dietary interventions, including only hypocaloric diets, on depression but not anxiety in adults with various metabolic conditions. In contrast, isocaloric dietary interventions only were not associated with a beneficial effect on either depression or anxiety scores.

The key finding from this meta-analysis was that dietary interventions overall, had beneficial effects on depression scores in adults without clinical depression with various metabolic conditions. This outcome is in line with observational evidence, which has suggested that dietary interventions can improve depression in a range of participants, including high-risk populations with metabolic conditions [4,5,31]. A meta-analysis of 15 RCTs (45,826 participants) including healthy adults as well as those with comorbidities all without reported clinical depression, showed that all types of dietary interventions including improving nutrition (e.g., reducing SFA intake, increasing plant-based foods), reducing fat intake, and weight loss diets had significant beneficial effects on depression scores (hedges = 0.246; 95% CI: 0.07, 0.423, *P* = 0.006) [31]. This meta-analysis applied no restrictions on diagnosis of depression or any other clinical or demographic characteristics, which provides further evidence to support our findings that dietary approaches can improve depression across a wide spectrum of the population. It is also worth noting that the small albeit significant effect size (SMD = −0.20) in our meta-analysis in those with metabolic conditions, was comparable with this previous meta-analysis in a broader, healthy population that reverse-coded the effects (hedges = 0.246), where a positive hedges score represents a beneficial intervention effect [31]. For our study, we cannot determine which dietary recommendations are most effective for improving depression in adults with metabolic conditions as the studies included heterogenous dietary recommendations. However, the general aim and intent of most dietary interventions hold common features including increasing plant-based foods such as vegetables and fruits; increasing healthy fats; and reducing intake of takeaway foods, refined CHO, and SFAs from “junk” food. Plant-based foods and healthy fats contain bioactive compounds (e.g., vitamins, minerals, fiber, polyphenols, and fatty acids), which are recommended to improve metabolic pathways including inflammation, insulin resistance, mitochondrial dysfunction, and oxidative stress, all of which have been linked with lower rates of depression and metabolic conditions [3,[55], [56], [57], [58]]. However, 7 of these 13 studies in our primary meta-analysis were hypocaloric, which implies that they were targeting weight loss that is known to have a positive effect on depression.

Weight loss (mainly from lifestyle interventions) has demonstrated the benefits for depression in adults with obesity [[59], [60], [61]], however, there is limited research looking at only hypocaloric diets on depression [30,31]. Another important finding from our meta-analyses was that in those who had metabolic conditions, hypocaloric diets designed and aiming to elicit weight loss significantly improved depression scores. There appeared to be a small yet significant additional benefit with hypocaloric dietary interventions compared with all dietary interventions (SMD = −0.27 compared with −0.20). Therefore, weight loss from hypocaloric diets may have been driving and mediating the overall relationship between all dietary interventions and depression. Of the 7 hypocaloric diet studies in our meta-analysis, half of which reported a significant improvement in depression scores [46,[50], [51], [52]], all reported significant weight loss in favor of the dietary intervention, with the magnitude of benefits ranging from a mean within-group loss of 1.1–9.7 kg in 5 studies [45,[48], [49], [50],^52^], 3.3 kg/m^2^ for BMI in another trial [51], and a mean difference in weight loss between groups of 2.95 kg [46]. This is in line with a previous systematic review (no meta-analysis) of 16 studies, which reported that calorie-restricted diets (10 of which had reported and led to a within-group mean weight loss ranging from 1.1 to 13.7 kg) were associated with improvements (effect size between ≈0.2 and ≈0.6) in depressive symptoms in participants who were overweight or obese [30]. Although loss of total body weight outcomes were not investigated in our results, research has shown that a 5%–10% loss in total body weight can improve metabolic risk factors (e.g., T2DM and hypertriglyceridemia) [62,63]. These improvements are hypothesized to be a consequence of improved inflammation, oxidative stress, and mitochondrial dysfunction [64,65], all factors which are also known to influence depression [3,56,[66], [67], [68]]. Therefore, although weight loss has been shown to improve depression, the exact magnitude of loss required to elicit improvements has not yet been established. Besides improvements in metabolic pathways, improvements in social factors, for example, weight-based stigma, may have also played a role in improving depression [69]. Although weight-based stigma outcomes were not reported in our results, a prior meta-analysis with 30 studies reported an association with depression and anxiety [69]. Therefore, it is plausible that weight loss may have improved weight-based stigma, which may have played a positive role in depression. Our results suggest that although dietary interventions overall are beneficial for depression outcomes, hypocaloric diets tended to result in a slightly more favorable effect as weight loss may have been the mediator driving the positive relationship in overall dietary interventions on depression.

Our findings also demonstrated that when isocaloric diets were assessed in isolation in adults with metabolic conditions, there were no beneficial effects on depression scores, which seems to conflict with existing literature from RCTs looking at plant-based diets (e.g., Mediterranean diet) aimed at improving depression in those with clinical depression [[70], [71], [72], [73]]. It is difficult to isolate the main reason to explain conflicting findings between our findings and current research. However, there may be several reasons such as there were many differences between each of the 6 included studies in the dietary interventions, length of intervention, and primary aims and results which is demonstrated by the *I*^2^ result of 69% defined as moderate heterogeneity between studies found to be a significant result (*P* = 0.004). Existing literature exploring isocaloric diets has mostly been conducted and reported positive findings in studies where depression was the primary aim [[70], [71], [72], [73]]. Only 2 of 6 studies in our meta-analysis had depression as the primary outcome and found significant between-group differences in depression in favor of the dietary intervention group [42,53]. There were also a large variety of dietary intervention topics (e.g., the Mediterranean diet [44]; low-fat, SFA, and cholesterol [47]; low-fat vegan [42]; food and nutrients for the microbiome [53]; Mediterranean DASH diet [54], and following a plant-based diet [43]), with the duration of studies ranging between 1-mo [54] and 3 y [43], contact time with the dietitian ranging between weekly [42,54] to 6 mo [43], and differences in the assessment and definition of dietary adherence and/or intake to the dietary intervention (e.g., Mediterranean DASH intervention for neurodegenerative delay score [54], 3-d food diary [49], 24-h recall [50]), to determine what had the greatest impact on depression. Therefore, further clinical trials that are adequately powered and with similar methodology are required to establish whether isocaloric dietary interventions are effective at improving depression.

Previous research demonstrates that those with clinical anxiety experience improvements in anxiety scores with improved diet quality (e.g., increasing vegetables, oily fish, fruit, whole grains) and reduced intake of discretionary foods (e.g., high-fat and sugar and processed foods) [55,74]. The findings from our meta-analyses show that that all dietary interventions, including isolating only hypocaloric and isocaloric interventions had no beneficial effect on anxiety scores in adults with metabolic conditions. We had a small number of studies (*n* = 7), 2 of which were hypocaloric and 5 of which were isocaloric included in our meta-analyses, which may explain the very small effect sizes (−0.05, −0.06, and −0.04, respectively) and no beneficial effect on anxiety. Our findings are consistent with 3 previous meta-analyses in nonclinical cases of anxiety that found that all dietary interventions (*n* = 11; hedges = 0.100, 95% CI: −0.04, 0.24, *P* = 0.148), weight loss (*n* = 4; hedges = 0.058, 95% CI: −0.067, 0.183, *P* = 0.366) and isocaloric-only diets (*n* = 6; hedges = 0.397, 95% CI: −0.173, 0.967, *P* = 0.173) were not effective at improving anxiety [31]. Multimodal lifestyle interventions (e.g., diet, exercise, and stress management) may be required rather than diet alone, to have a greater impact on anxiety outcomes. Although no studies to date have assessed whether multimodal lifestyle interventions are superior to diet alone for anxiety outcomes, evidence shows that lifestyle interventions do improve anxiety outcomes [60,75,76]. For instance, a meta-analysis of 4 RCTs assessing the effect of lifestyle interventions compared with a control group on anxiety levels among 148 females, reported a pooled estimate (SMD = −1.74; 95% CI: −2.62, −0.87, *P* < 0.001), which compared with our pooled result of −0.05, suggests there may be a superior effect of lifestyle compared with dietary-only interventions [59]. Therefore, further adequately powered clinical trials with well-designed, comprehensive, and tailored dietary interventions are required to determine if dietary interventions are effective at improving anxiety in adults with metabolic conditions.

There are multiple strengths in this systematic review and meta-analysis. The study design was comprehensive with the review conducted in line with the most current PRISMA guidelines [35]. The eligibility was robustly designed to capture dietary interventions alone to answer the research question. Furthermore, there were several meta-analyses conducted that enabled us to answer our primary aim of assessing the effect of dietary interventions on depression and anxiety in people who have metabolic conditions, as well as determine the effects of hypo- and isocaloric diets in isolation.

Despite its strengths there are several limitations that need to be acknowledged in this systematic review and meta-analysis and also in this field of research as it is poorly investigated. First, depression and anxiety were secondary outcomes for most of the included studies, and therefore studies were not powered to detect changes or group differences in depression and anxiety. Furthermore, studies did not recruit participants with depression and anxiety at baseline and thus it is likely that there was limited capacity for improvement as low baseline levels reduce the scope for change. Furthermore, based on risk of bias assessment, 10 studies obtained an overall result of “poor quality” and 3 studies obtained “fair quality,” which could impact the validity of the results. Furthermore, depression and anxiety symptoms were measured with a variety of questionnaires, making comparison of results inherently challenging. Although all the studies used validated tools to measure depression and anxiety, they were not validated within populations with metabolic conditions, which is a further limitation. There was also a moderate level of heterogeneity measured using the *I*^2^ statistic in the meta-analyses assessing all dietary and only isocaloric in depression and anxiety outcomes, and there was a large variation in the type of dietary interventions, contact time with dietitians/researchers, and length of the interventions. However, due to the small number of studies included, it was not considered appropriate to explore sources of heterogeneity using meta-regression analysis. Finally, studies lacked the ability to define dietary adherence, whereas other studies had different methods to measure dietary intake and others did not even report dietary intake.

In conclusion, our findings suggest that dietary interventions overall and hypocaloric diets only, can improve depression but not anxiety in people with metabolic conditions. In contrast, there were no beneficial effects of isocaloric dietary interventions on either depression or anxiety. This suggests that dietary interventions may be an effective strategy for managing depression in adults with metabolic conditions. Caution is warranted when interpreting results due to the overall low quality and small effect size of the studies included. Future clinical trials should consider depression and anxiety as the primary outcome, measure and define dietary adherence, use validated tools in participants with metabolic conditions, and also explore specific dietary patterns including isocaloric and/or hypocaloric diets that are comprehensively designed and measured.

## Author contributions

The authors’ responsibilities were as follows – TP: designed and conducted research, conducted database searches, and contributed to the primary responsibility of the final content; TP, CLF, and SS: conducted screening of the title, abstract, and full text; ESG: resolved conflicts of studies in database searches; TP and GA: analyzed data and conducted all statistical analysis; TP: wrote the manuscript with feedback; ESG, RMD, MCR, and GA: provided suggestions and edits; and all authors: read and approved the manuscript.

## Conflict of interest

All authors have no conflicts of interest.

## Funding

TP was supported by a Deakin University, Faculty of Health Doctor of Philosophy scholarship. ESG was supported by a Deakin University, Faculty of Health Dean’s Postdoctoral Fellowship.

## Data availability

Data described in the manuscript will be made available upon request pending (e.g., emailing the corresponding author).
