# Supplementary material for: Diet Overall and Hypocaloric Diets Are Associated With Improvements in Depression but Not Anxiety in People With Metabolic Conditions: A Systematic Review and Meta-Analysis
Source: Adv Nutr. 2024 Jan 5;15(2):100169. doi: 10.1016/j.advnut.2024.100169 (PMC10847486; doi:10.1016/j.advnut.2024.100169)
Supplement: Multimedia component 1 [file mmc1.docx]

**
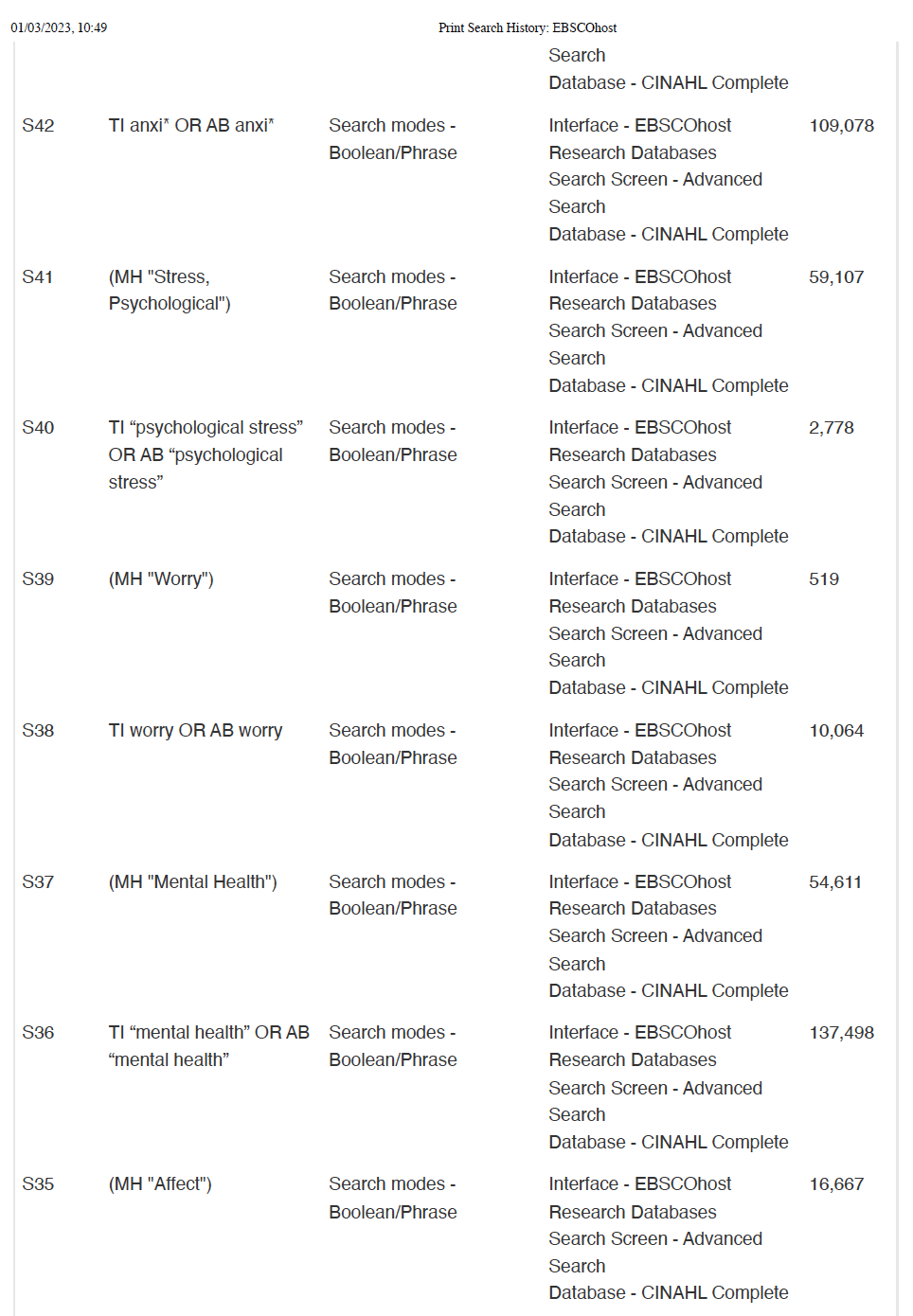
**

**
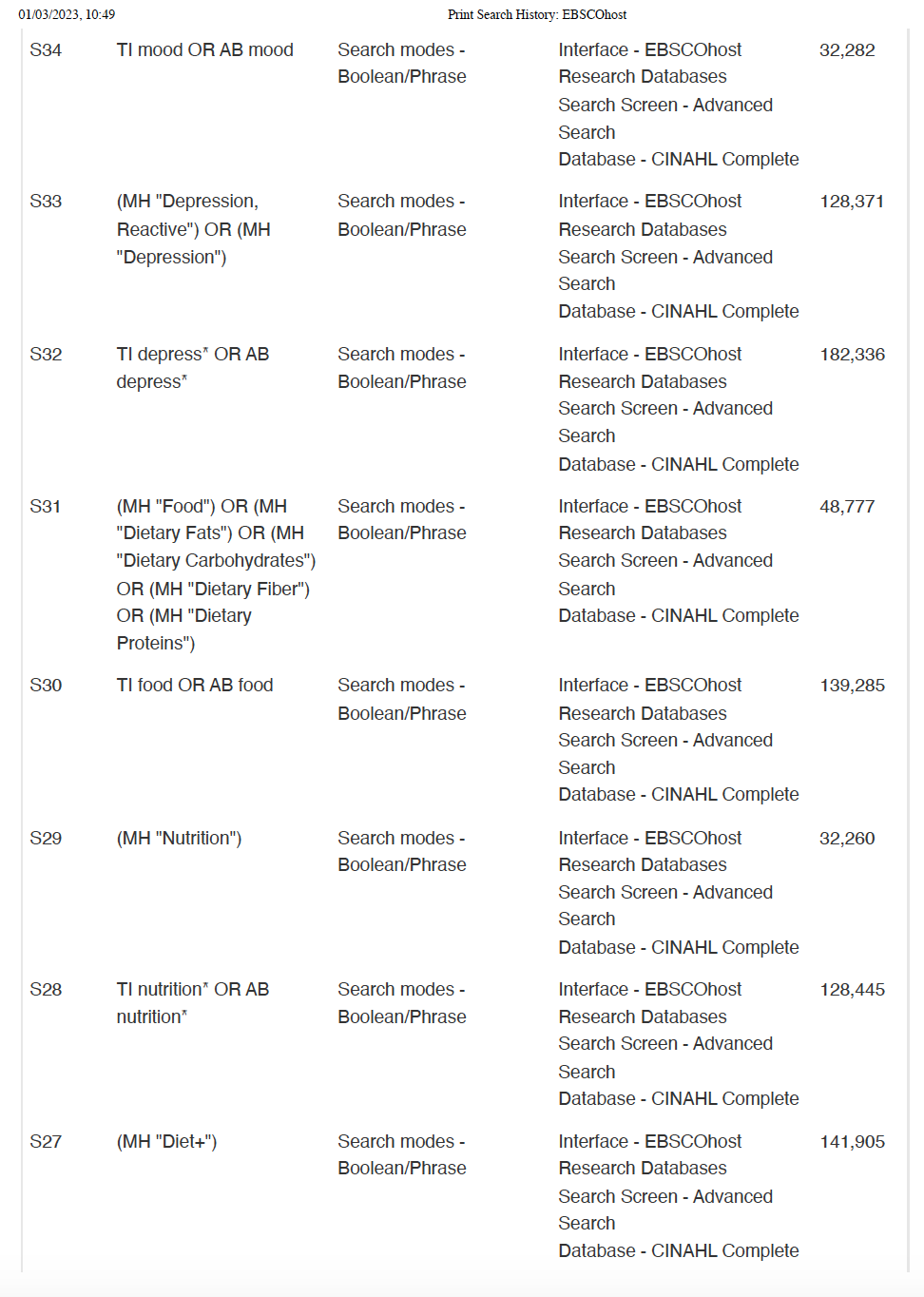
**

**
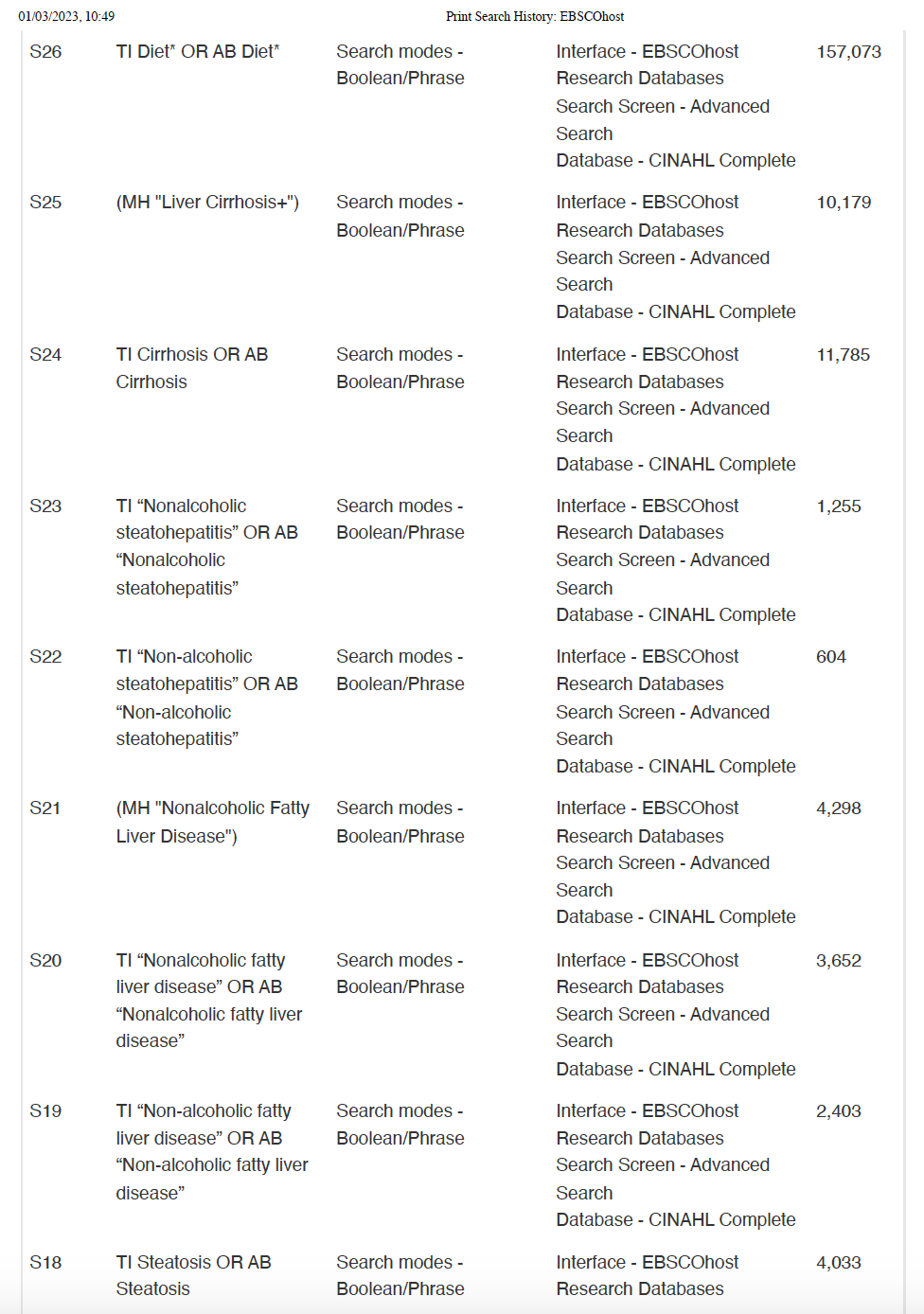
**

**
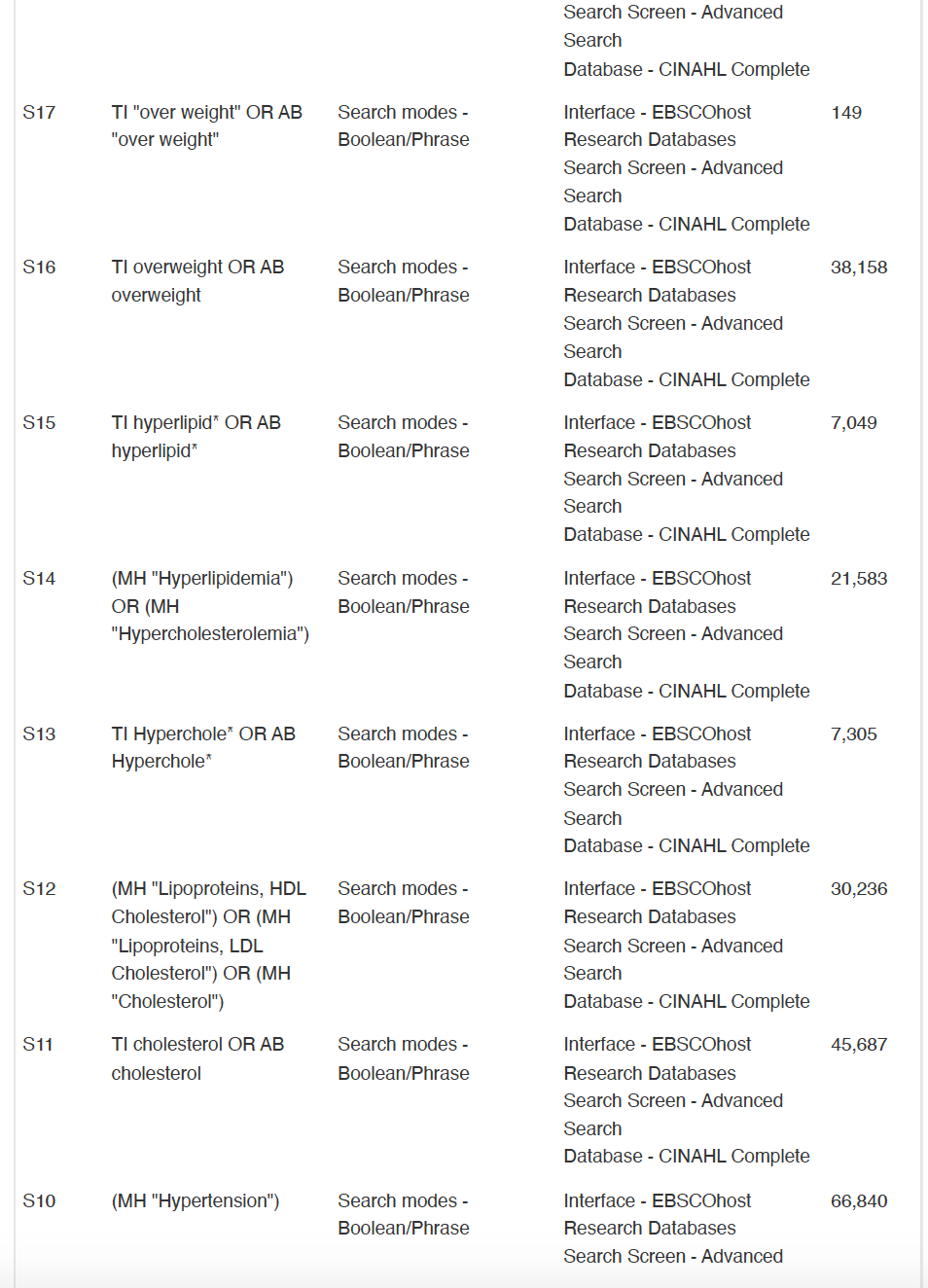
**

**
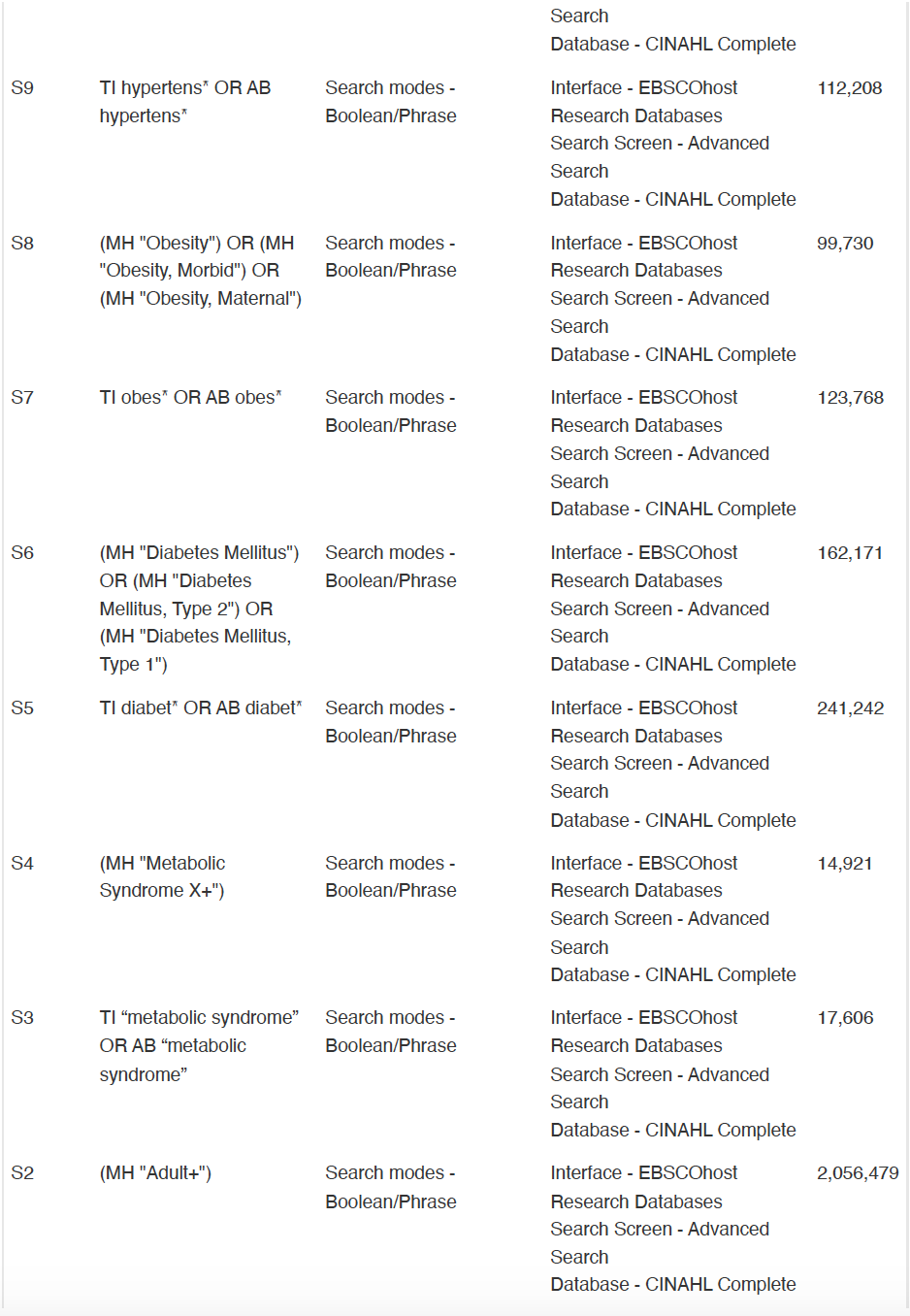
**

**
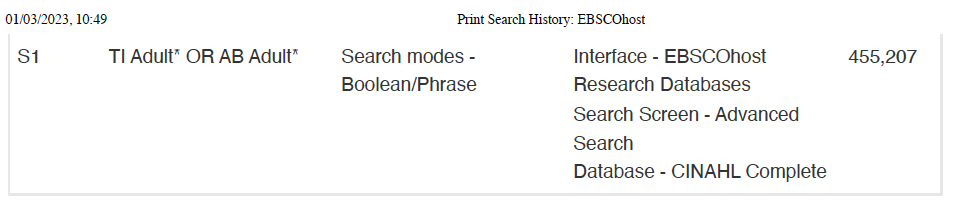
**

**Supplemental Figure 1.** Searches for Cinahl database


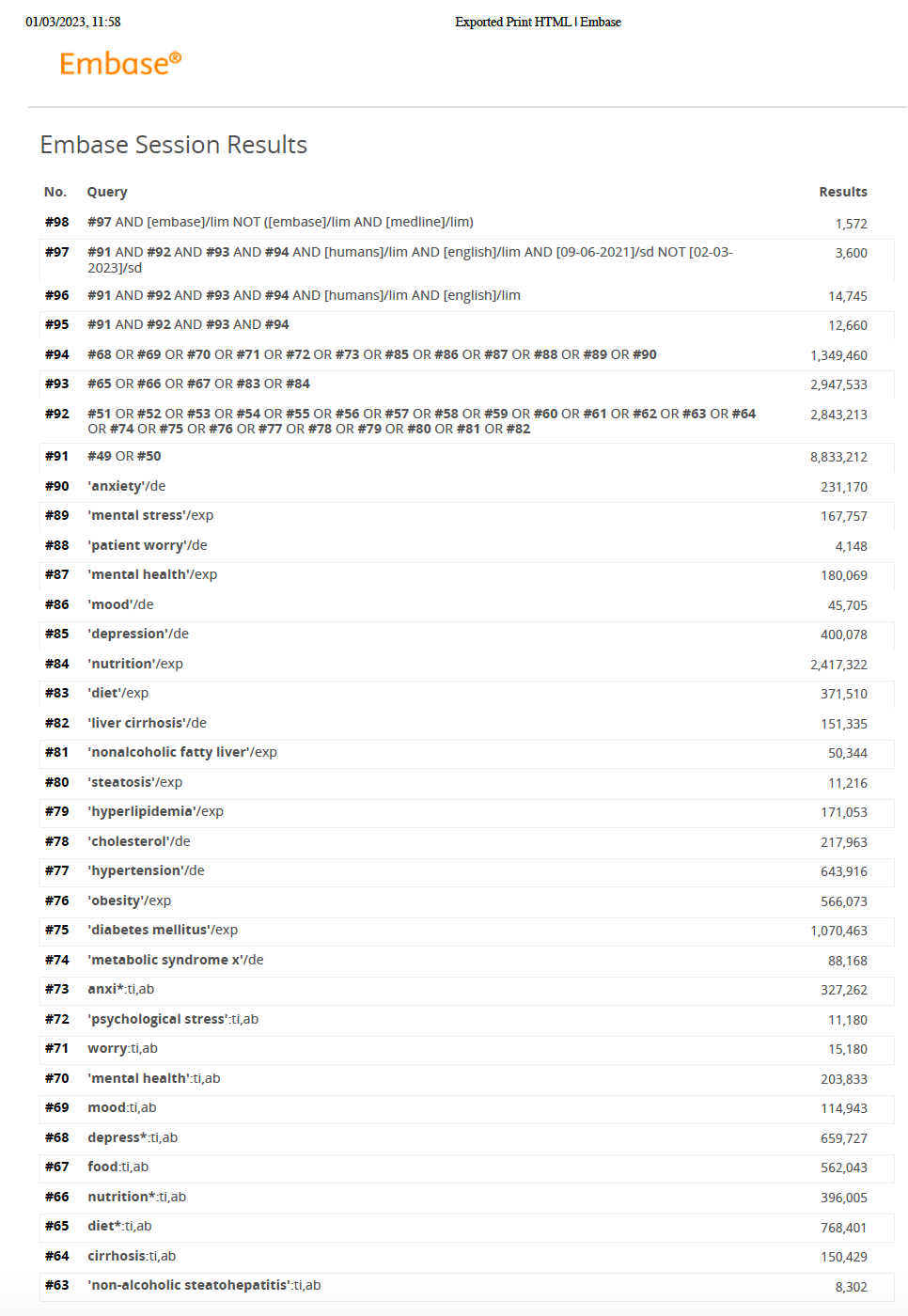


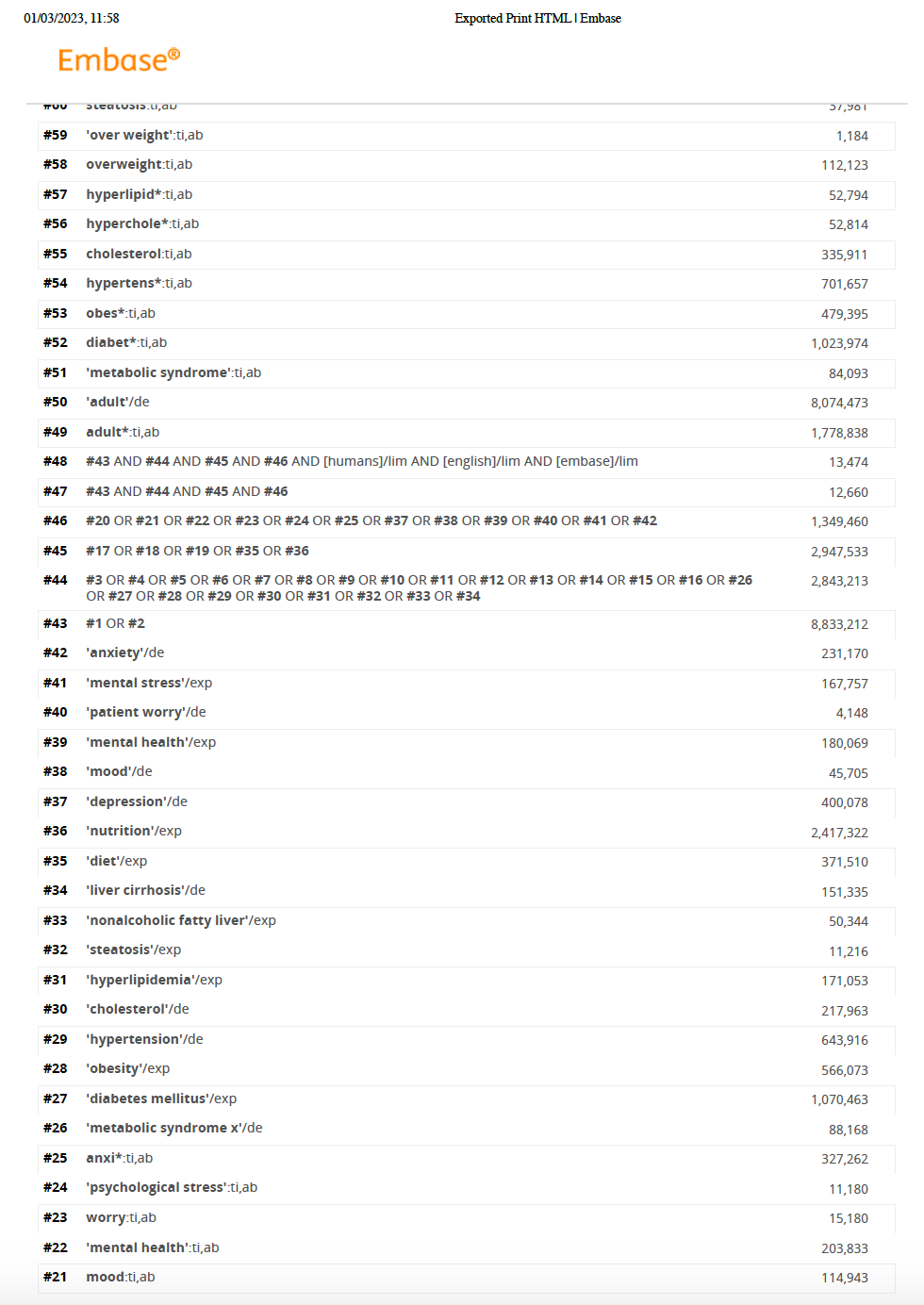


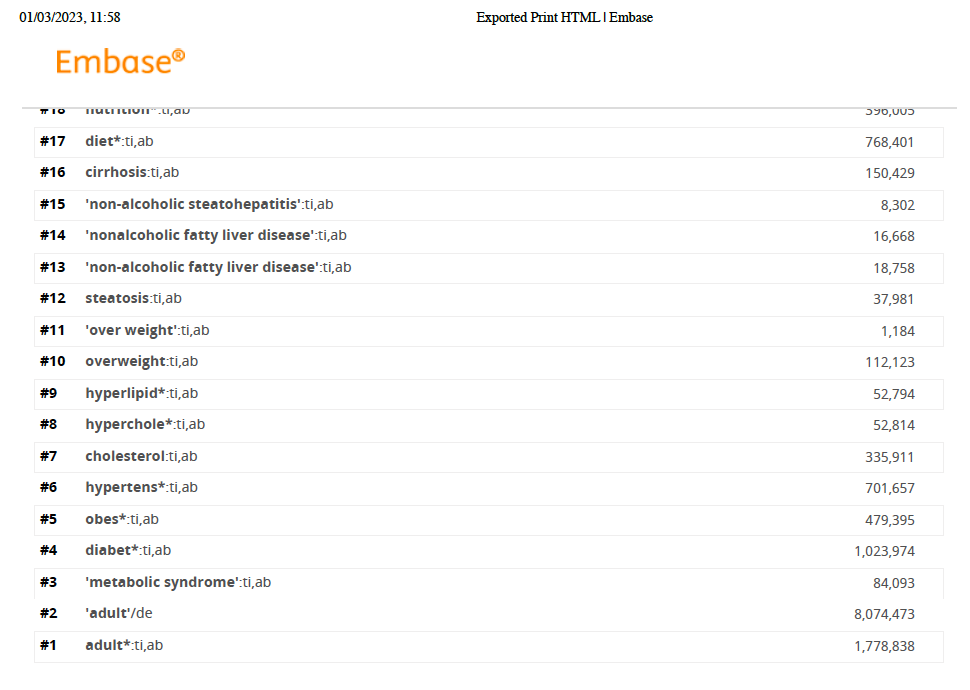


**Supplemental Figure 2.** Searches for Embase database
