# Supplementary material for: Diet Overall and Hypocaloric Diets Are Associated With Improvements in Depression but Not Anxiety in People With Metabolic Conditions: A Systematic Review and Meta-Analysis
Source: Adv Nutr. 2024 Jan 5;15(2):100169. doi: 10.1016/j.advnut.2024.100169 (PMC10847486; doi:10.1016/j.advnut.2024.100169)
Supplement: Multimedia component 4 [file mmc4.docx]

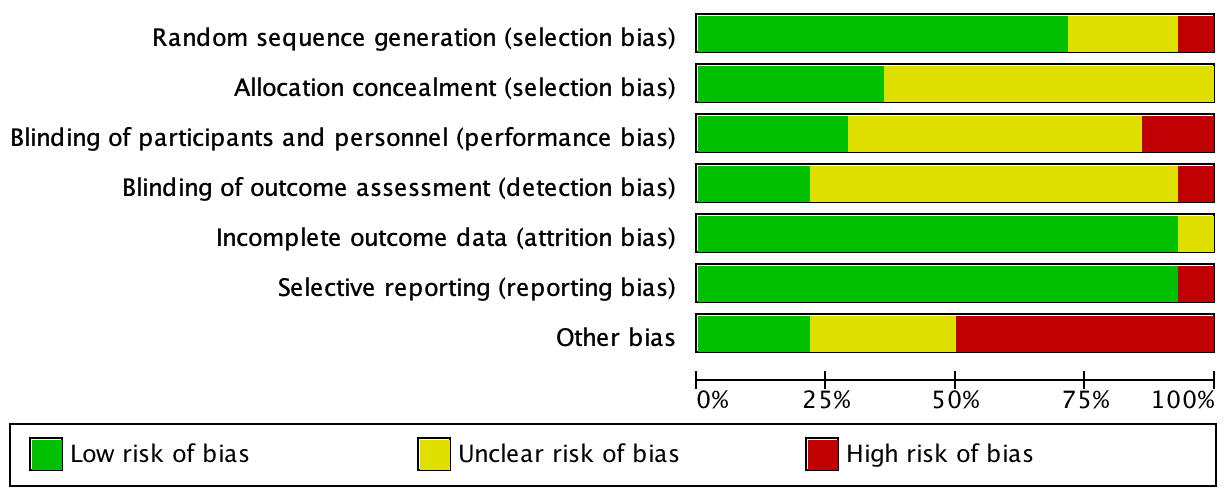


**Supplemental Figure 5.** Risk of bias graph.


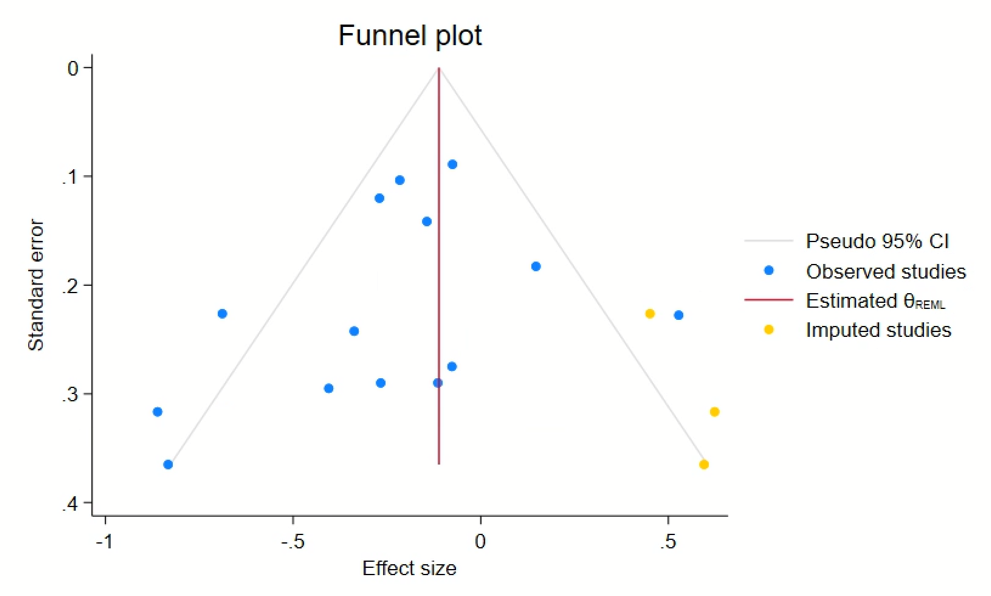


**Observed studies=**-0.219 (95% CI -0.373 to -0.47).

**Observed + Imputed=**-0.112 (95% CI -0.301 to 0.078).

Observed studies 14, imputed 3.

**Supplemental Figure 6.** Funnel plot of the effects of dietary interventions on depression scores.


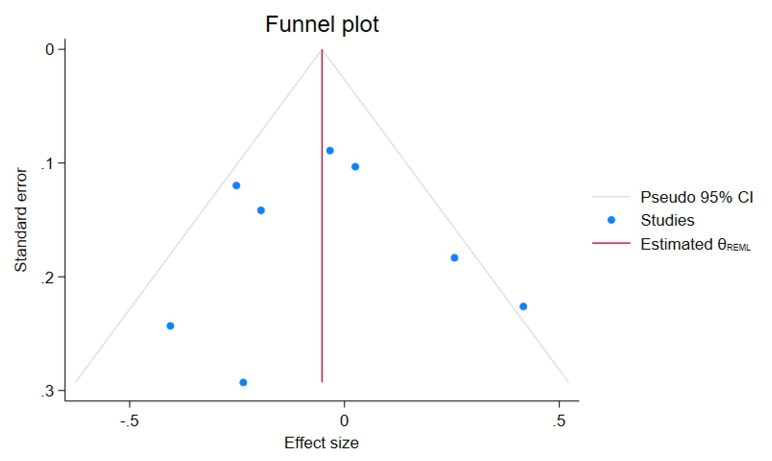


**Observed studies=**-0.052 (95% CI -0.200 to 0.096).

Observed studies 8.

**Supplemental Figure 7.** Funnel plot of the effects of dietary interventions on anxiety scores.

**
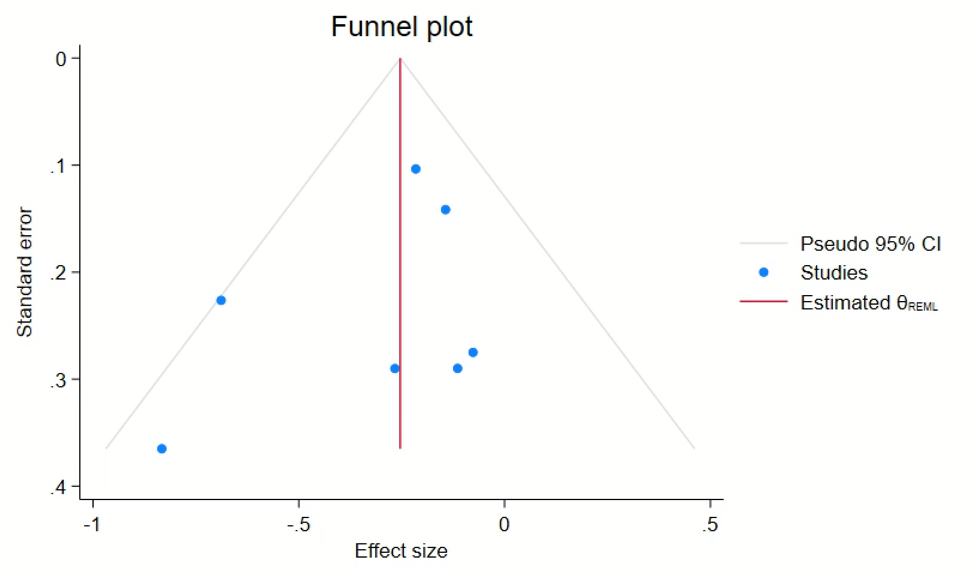
**

**Observed studies=**-0.254 (95% CI -0.390 to -0.117).

Observed studies 7.


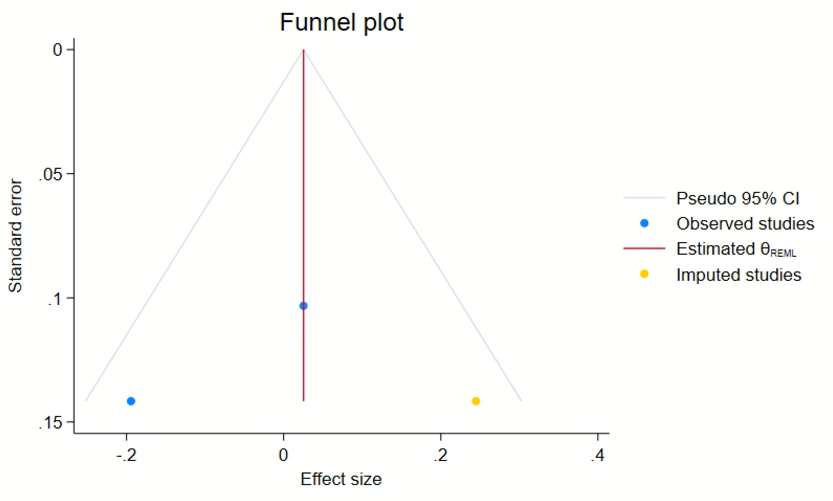
**Supplemental Figure 8.** Funnel plot of the effects of hypocaloric dietary interventions on depression.

**Observed studies=**-0.063 (95% CI -0.274 to 0.148)

**Observed + Imputed=**0.025 (95% CI -0.204 to 0.254)

Observed studies 2, imputed 1.

**Supplemental Figure 9.** Funnel plot of the effects of hypocaloric dietary interventions on anxiety.


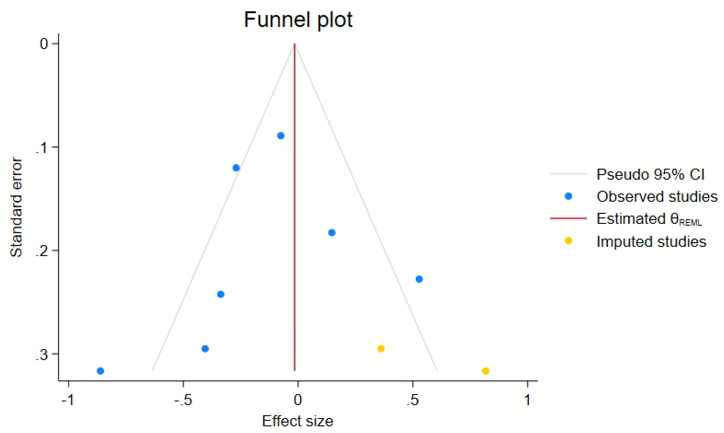


**Observed studies=**-0.146 (95% CI -0.430 to 0.139).

**Observed + Imputed=-**0.015 (95% CI -0.319 to 0.289).

Observed studies 7, imputed 2.
**Supplemental Figure 10.** Funnel plot of the effects of isocaloric dietary interventions on depression.


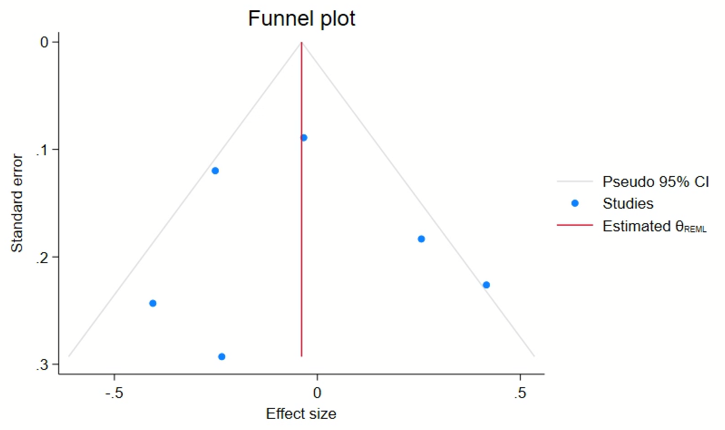


**Observed studies=**-0.039 (95% CI -0.268 to 0.190).

Observed studies 6.

**Supplemental Figure 11.** Funnel plot of the effects of isocaloric dietary interventions on anxiety.
