# Supplementary material for: Diet Overall and Hypocaloric Diets Are Associated With Improvements in Depression but Not Anxiety in People With Metabolic Conditions: A Systematic Review and Meta-Analysis
Source: Adv Nutr. 2024 Jan 5;15(2):100169. doi: 10.1016/j.advnut.2024.100169 (PMC10847486; doi:10.1016/j.advnut.2024.100169)
Supplement: Multimedia component 5 [file mmc5.docx]

**Supplemental Table 1.** Dietary adherence and/or dietary intake and changes in depression scores, anxiety scores and weight for the 13 dietary intervention studies included in the systematic review and meta-analysis.

| **Citation** | **Dietary adherence and/or dietary intake** | | **Between group differences** | | **Within group change** |
| --- | --- | --- | --- | --- | --- |
| Agarwal 2015 | Two 24-hour recalls at baseline and two 24-hour recalls at week 18 using online automated self-administered 24-hour recall program. Cholesterol intake was used to measure adherence to the vegan diet. Total and SFA intakes was used as a measure of adherence to the low-fat diet. Cholesterol intake at 18 weeks was ≤75 mg/day in 85% of the diet group and 21% of the control group (p <0.001). Cholesterol intake was ≤50 mg/day for 74% of the diet group and 13% of the control group (p<0.001). Total fat intake was ≤35% of the total energy intake in 86% of the diet group compared with 40% of the control group (p<0.001). Fat intake was ≤25% for 49% of the diet group and for 8% of the control group (p<0.001). Saturated fat intake was ≤10% of total energy intake in 88% of the diet group and 38% of the control group (p<0.001). Saturated fat intake was ≤5% of total energy intake for 51% of the diet group and 5% of the control group (p<0.001). Both groups reduced their energy intake, but the between-group difference was not significant. | | Depression:  -5.17 (95% CI -8.63 to -1.72) (p<0.001) (unadjusted)  3.72 (0.49 to 6.94) (adjusted^1^)  Anxiety:  -4.67 (-8.24 to -1.72) (p<0.05) (unadjusted)  3.59 (0.25 to 6.92) (adjusted^1^)  Weight not reported | | **Diet group**  Depression:  -4.80 ± SD 15.04 (p<0.001)  Anxiety:  -5.22 ± 15.87 (p<0.001)  **Control group**  Depression:  0.37 ± 14.32  Anxiety:  -0.552 ± 14.42  Weight not reported |
| Einvik 2010**^2^** | Intake was measured using FFQ at baseline and after 3 months.  There was a significant between group difference in SFA intake (diet group: 27.0 ± SD 10.4g/d to 21.4 ± 8.3g/d, no diet group: 27.2 ± 9.9 g/d to 24.2 ± 8.5g/d), p<0.001^3^), percentage E intake from CHO (diet group: 49.1 ± 6.6% to 51.7 ± 6.8%, no diet group: 48.6 ± 6.1% to 50 ± 6.4%, p=0.001^3^), percentage E intake from fat (diet group: 30.4 ± 5.4% to 27.6 ± 5.5%, no diet group: 31.3 ± 5.5% to 29.5 ± 5.4%, p<0.001^3^), MUFA (diet group: 26 ± 8.7g/d to 18.4 ± 6.3g/d, no diet group: 27 ± 8.1g/d to 20.1 ± 6.4 g/d, p=0.007^3^), PUFA (diet group: 12.5 ± 5g/d to 13.1 to ± 5.5g/d, no diet group: 13.8 ± 5g/d to 12.7 ± 4.9g/d, p=0.045^3^), omega 3 (diet group: 2.8 ± 1.2g/d to 2.7 ± 1.1g/d, no diet group: 2.9 ± 1.3 to 2.5 ± 1.2g/day, p=0.012), PUFA:SFA ratio (diet group: 0.49 ± 0.16 to 0.63 ± 0.17, no diet group: 0.53 ± 0.17 to 0.55 ± 0.16, p<0.001) and fibre intake (diet group: 21.6 ± 7g/d to 22.4 ± 6.9g/d, no diet group: 22 ± 7.5g/d to 20.4 ± 6.8g/d, p<0.001^3^). There was no significant between or within group change for energy, fat grams, protein, omega 6 and α-linolenic acid and CHO grams intake^3^. | | No significant differences in depression and anxiety.  No significant differences in weight loss^4^. | | **Diet group** *n*=253  Depression:  **Baseline**  3.4 ± SD 2.7  **Post**  4.1 ± 2.8 (p<0.001)  Anxiety: **Baseline**  3.2 ± 2.6  **Post**  3.9 ± 3.0 (p<0.001)  **No diet group** *n*=252  Depression: **Baseline**  3.7 ± 2.8 **Post**  4.3 ± 2.5 (p<0.01)  Anxiety:  **Baseline**  3.4±2.7 **Post**  4.0 ± 2.9 (p<0.01)  **Diet group and placebo weight (BMI):**  **Baseline** 26.8 ± 3.8kg/m^2^ **Post**  26.6 ± 4kg/m^2^  **Control group weight (BMI) Baseline**  26.6 ± 3.4kg/m^2^  **Post** 27 ± 3.9kg/m^2^  (significance not reported) |
| Hyyppä 2003 | Seven-day food diary at baseline and week 12. There was a significant within group reduction in cholesterol (298 ± SD 92 mg/d to 214 ± 82mg/d), percentage E intake from SFA (13.5 ± 5% to 9.3 ± 2.1%) in the diet and placebo group (p<0.001). There was a significant increase in MUFA (11.8 ± 1.7% to 14.1 ± 3%), PUFA (5.7 ± 1.2% to 8.1 ± 1.6%), linolenic (1.01 ± 0.35g/d to 3.57 ± 1.45g/d) and linolic acid (6.68 ± 2.53g/d to 11.45 ± 3.59g/d), fibre (20 ± 6.7g to 27.2 ± 7.8g), Vitamin C (62 ± 38mg/d to 93 ± 46mg/d), and vitamin E (9.5 ± 2.9mg/d to 14.6 ± 4mg/d) intake in the diet and placebo group (p<0.001). No change in percentage of E intake from fat and b-carotene in the diet and placebo group. No change in diet intake in the control and placebo group, besides a significant within group reduction in b-carotene (2453 ± 1580μg/d to 1693 ± 1100μg/d) (p<0.001). | Not reported | | **Diet group**  Depression:  0.03 ± SEM 0.04  Anxiety:  0.05 ± 0.03  Weight:  **Baseline**  82.4kg ± SD 9.3  **Post (placebo)**  82.7kg ± 9.5 (Significance not reported)  **Control group** Depression and anxiety not reported Weight:  **Baseline**  81.2kg ± 9.7  **Post (placebo)**  82.1kg ± 9.6 (Significance not reported) | |
| Imayama 2011 | Measured using a FFQ at baseline and at one-year. Both groups reduced calorie intake (diet group: 1884 ± SD 661kcal/d to 1637 ± 621kcal/d, control group: 1988 ± 669kcal/d to 1768 ± 606kcal/d) with no significant difference between groups. There was a significant greater reduction in percentage of E intake from fat in the diet group (33.1 ± 6.3% to 27.3 ± 7.2%) compared to the control group (35.6 ± 6.9% to 33.4 ± 6.8%) (p<0.0001). No within group statistics was reported. | | No significant difference in depression and anxiety^5^  Significant difference in weight loss (p<0.0001) | | **Diet group** Depression: **Baseline** 49.4 ± SD 9.8 **Post** 47.8 ± 8.7 (Unadjusted) Mean changes -0.5 SD not reported (adjusted**^6^**) Anxiety:  **Baseline** 44.4 ± 6.8 **Post** 43.8 ± 7.3 (Unadjusted) Mean changes -0.6 SD not reported (adjusted**^6^**) Weight: **Baseline** 84.0kg ± 11.8  **Post** 76.9kg ± 13.4 (Significance not reported)  **Control group** Depression: **Baseline** 48 ± 9 **Post** 48.4 ± 9.6  (Unadjusted) Mean changes 0.7 SD not reported (adjusted**^6^**) Anxiety: **Baseline** 45.3 ± 7 **Post** 45.3 ± 8.7 (Unadjusted) Mean changes 0.6 SD not reported (adjusted**^6^**) Weight: **Baseline** 84.2kg ± 12.5 **Post** 83.5kg ± 12.3 (Significance not reported) |
| Jenkinson 2009 | Not reported. | | Not reported in depression and anxiety  The mean difference in weight loss in the diet group compared with no diet group was 2.95kg (95% CI 1.44 to 4.46) (p=0.000) | | **Diet group** *n*=231  Depression:  −0.67 ± SE 0.32 (p=0.037)  Anxiety:  0.09 ± 0.35  **Exercise group** *n*=191  Depression:  0.15 ± 0.37  Anxiety:  −0.26 ± 0.37  Weight not reported. |
| Kiernan 2001 | Seven-day food diary at baseline and at one-year. The diet group had a significant greater reduction in energy (males in diet group: -2915 ± SD 2577kJ/d, males in control group: 155 ± 2627kJ/d, females in diet group: -2180 ± 1531kJ/d, females in control group: 60 ± 1800kJ/d), percentage of E intake from fat (males in diet group: -6 ± 6.8%, males in control group: 0.8 ± 5.2%, females in diet group: -7.8 ± 8.2%, females in control group: -0.9 ± 5.7%), SFA (males in diet group: -3.2 ± 2.9%, males in control group: -0.2 ± 2.3%, females in diet group: -3.7 ± 3.7%, females in control group: -0.4 ± 3.3%), and cholesterol intake (males in diet group: -159 ± 171mg/d, males in control group: 7 ± 145mg/d, females in diet group: -123 ± 98mg/d, females in control group: -5 ± 88mg/d), compared to the control group (p<0.001). | | Not reported in depression and anxiety  Significant difference in weight loss (males and females) (P<0.001) | | **Diet group**  Depression:  1.1 ± SD 3.8 (males)  -1.4 ± 4.4 (females) Anxiety:  1.3 ± 3.0 (males)  -0.5 ± 2.7 (females)  Weight:  -5.0kg ± 5.8 (p<0.001) (males)  -4.1kg ± 5.5 (p<0.001) (females)  **Control group**  Depression:  -0.7 ± 2.9 (males)  0.3 ± 5.4 (females)  Anxiety:  0.1 ± 2.7 (males)  0.8 ± 3.5 (females)  Weight:  1.7kg ± 4.8 (males)  1.3kg ± 5.2 (females**)** |
| Napoli 2014 | Food diaries were reviewed; food intake or adherence was not reported. | | No significant difference in depression  0.2 (95% CI -1.0 to 1.3)  Significant difference in weight loss (p<0.001) | | **Diet group**  Depression:  -0.2 ± SD 0.3  Weight:  -9.7kg ± 5.4 (p<0.001)  **Control group**  Depression: -0.1 ± 0.4  Weight: -0.1kg ± 3.5 |
| Nieman 2000 | At baseline 3-day food diary and 11 random 24-hour dietary recalls during the study. At baseline intake was 8.63 ± SE 0.32MJ/d and 7.88 ± 0.35MJ/d for the groups randomized to diet and exercise + diet, and control and exercise, respectively. Post intervention energy intake was 5.31 ± 0.16MJ/d for the diet and exercise + diet groups, with percent of E intake as CHO, fat, and protein measured at 60.3 ± 0.9%, 22.3± 0.7%, and 19.1 ± 0.3%, respectively. Before and during the intervention, mean intake of vitamins and minerals (vitamins C, E, A, B6, B12, folate, iron, selenium) exceeded 67% of the recommended dietary allowance for the two diet groups (diet and diet + exercise) and two non-diet groups (control and exercise). Statistical significance was not reported. | | No significant difference in depression  Significant difference in weight loss (p<0.05) | | **Diet group**  Depression: **Baseline**  120 ± SE 6  **Post**  114 ± 7  Weight: **Baseline** 90.6kg ± 3.6  **Post**  82.8kg ± 3.7 (p<0.05)  **Control group** Depression:  **Baseline**  120 ± 6  **Post**  118 ± 7  Weight:  **Baseline**  90.5kg ± 2.4  **Post**  89.7kg ± 2.5 |
| Ozbey-Yucel 2023 | Twenty-four hour recall at baseline and at week 12. The diet group had a significant greater reduction in calories (diet group: -205.7 ± SD 177.2kcal, control group: -74.6 ± 19.9kcal, p=0.001), CHO (diet group: -21.3 median IQR 19.7g, control group: -7.6 median IQR 7.5g, p=0.001), total fat (diet group: -12.1 median IQR 16.3g, control group: -2.0 median IQR 6.3g, p=0.001), SFA (diet group: -2.3 median IQR 6.6g, control group: -1.2 median IQR 1.6g, p=0.001), PUFA (diet group: -8.9 median IQR 10.6g, control group: -2.0 median IQR 3.9g, p=0.009) and protein intake (diet group: -5.9 ± 7.7g, control group: -3.2 ± 5.6g, p=0.02) compared to the control group. No significant within and between group difference in MUFA. | | Significant difference in depression (p<0.001)  Significant difference in weight loss (p<0.001) | | **Diet group**  Depression:  -3.5 ± SD 1.0 (p<0.01)  Weight: median and (IQR)  -3.4kg (0.9) (p<0.01)  **Control group** Depression: -2.3 ± 1.7 (p<0.05)  Weight: median and (IQR)  0.3kg (0.6) |
| Senna 2012 | Participants were instructed on accurate recording of dietary intake; however, dietary data was not reported. | | Significant difference in depression  (95% CI 1.8431 to 7.8340) (p=0.002)  Significant difference in BMI (3.4952 to 4.5396) (p<0.001) | | **Diet group**  Depression:  **Baseline**  18.6 ± SD 8.7  **Post** 12.8 ± 5.8 (Significance not reported)  Weight (BMI): **Baseline**  32.3kg/m^2^ ± 1.4  **Post**  29.03kg/m^2^ ± 1.22 (p<0.001)  **Control Group**  Depression:  **Baseline**  17.9 ± 8.9  **Post**  17.6 ± 7.7  Weight (BMI): values not reported, no significant change. |
| Tan 2016 | Three-day food diary at baseline and 6 months. Energy intake was reduced at 6 months in the diet group (p=0.006), changes in other nutrients were not detected. Energy intake was reduced at 3 months in both groups (p=0.001 and 0.012, respectively). The diet group had greater intakes of potassium (2158 vs. 1806 mg/1000 kcal, p=0.029^7^) and magnesium (219 vs. 193 mg/1000 kcal, p = 0.036^7^) at 3 months compared to the control group. | | No significant difference in depression**^5^**  Significant difference in weight loss (p<0.05) | | **Diet group**  Depression:  **Baseline**  5.33 ± SD 2.34  **Post**  3.77 ± 3.67 (p=0.029)  Weight:  **Baseline**  93.8kg (95% CI 89.2 to 98.4)  **Post** 92.7kg (88.1 to 97.4) (p<0.05)  **Control group**  Depression:  **Baseline**  4.83 ± 3.58  **Post**  3.67 ± 2.39  Weight:  **Baseline**  93.1kg (85.2 to 100.9)  **Post** 94.4kg (86.3 to 102.5) (p<0.05) |
| Uemura 2019 | Brief-type dietary history questionnaire at baseline and week 8. There was a significant different between group, where the diet group showed increases in fibre (3.6g/day ± SD 4.6 vs -0.3g/d ± 4.6, p<0.01), vegetable dishes (2.4 serves/d ± 3.3 vs 0.5 serves/d ± 1.7, p=0.020), and milk products (1.5 serves/d ± 1.2 vs 0.4 serves/d ± 1.0, p<0.01) compared to the control group.  No significant between group difference in calories, CHO, total fat, protein, grain dishes, meat and fish, and fruit. | | Significant difference in depression (p<0.01)  Significant difference in weight loss (p<0.001)  (unadjusted and adjusted^8^) | | **Diet group**  Depression: − 7.59 ± SD 9.27 (Significance not reported)  Weight:  -1.69kg ± 1.35 (unadjusted)  -1.66kg ± 0.27  (adjusted^8^) (Significance not reported)  **Control group**  Depression: − 1.05 ± 5.03  (Significance not reported)  Weight:  -0.04kg ± 1.20 (unadjusted)  -0.07kg ± 0.27(adjusted^8^)  (Significance not reported) |
| Yau 2022 | The Mediterranean-DASH intervention for neurodegenerative delay score at baseline and after 1-month. A maximum score of 15 indicating the highest adherence. The mean score significantly increased by 3.8 in the diet group. Baseline and post intervention scores were not reported. FFQ at baseline only. Dietary intake was not reported. | | No significant difference in depression and anxiety -1.34 (95% CI -3.20 to 0.51), -2.27 (-7.69 to 3.15), respectively.  No significant difference in weight loss -0.86kg/m^2^ (-3.13 to 1.41) | | **Diet group**   Depression: 0.17 (95% CI -1.24 to 0.89) Anxiety:  -0.78 (-2.73 to 4.31)  BMI:  -0.04kg/m^2^ (-0.05 to 0.13)  **Control group**  Depression:  -0.12 (-1.21 to 1.46)  Anxiety:  -0.75 (-1.28 to 2.78)  BMI:  0.05kg/m^2^ (-0.24 to 0.14) |

**^1^**Adjusted for sex, cluster, medications, and baseline values in univariate analysis.
**^2^**In the study by Einvik 2010, the Oslo diet and Antismoking Study from 1972-1977 where all participants that received traditional lifestyle advice, including advice on cessation of smoking, and half the participants were randomized to dietary counselling (dietary counselling *n*=604 compared with no diet counselling *n*=628). The study relevant to this review is the 25 year follow up (DOIT) study with *n*=505 males participants from 1997 to 2003.
^3^Analysis of covariance with adjustment for baseline values.

4Analysis of covariance with adjustment for baseline values examining independent effects of diet.
^5^Adjusting for the baseline scores and covariates (Depression: medication use, Anxiety: medication use)
^6^Adjusted means are changes in psychological factors adjusted for baseline scores and covariates (e.g., age, baseline BMI, marital status, anxiolytics and antidepressants use)
**^7^**Controlling for baseline values

^8^Adjusted for energy intake as a covariate

CHO, carbohydrates; DASH, dietary approaches to stop hypertension; E, energy; FFQ, food frequency questionnaire; GI, glycaemic index; ITT, intention-to-treat.
